# Confined Chemical Transformation of Melamine in Graphite Interlayers Toward Graphite-Based Composites with Nitrogen-Rich Two-Dimensional Materials

**DOI:** 10.3390/nano16161028

**Published:** 2026-08-19

**Authors:** Wei Zhou, Haseeb Ur Rehman, Zeming Wang, Oleksandr Ivasenko

**Affiliations:** State Key Laboratory of Bioinspired Interfacial Materials Science, Institute of Functional Nano & Soft Materials (FUNSOM), Soochow University, Suzhou 215123, China; 20224214176@stu.suda.edu.cn (W.Z.); 20237114005@stu.suda.edu.cn (H.U.R.); 20244214026@stu.suda.edu.cn (Z.W.)

**Keywords:** graphite, melamine, ball milling, solid-state reaction, composites

## Abstract

Graphite-based nanocomposites with nitrogen-rich covalent two-dimensional materials are promising for energy, catalytic and sensing applications, but their controlled construction remains challenging because both graphite and many covalent 2D materials consist of stacked sheets that are difficult to integrate homogeneously without prior exfoliation, dispersion, mixing, and restacking. Here, we explore a solvent-free strategy that uses melamine-confined graphite as a preorganized precursor for chemical transformations between graphene layers. We demonstrate that intercalated melamine can undergo reaction pathways analogous to those of bulk melamine, enabling not only the previously reported formation of graphite/g-C_3_N_4_ composites but also the construction of a new graphite/melem composite. The same concept is further extended to multicomponent solid-state reactions by introducing pyromellitic dianhydride, enabling the formation of new graphite/polyimide-linked two-dimensional material composites from either melamine or melem precursors. Comparison of one-pot and stepwise routes shows that precursor preorganization within graphite improves framework preservation, structural continuity, and morphological homogeneity. Overall, this work presents graphite interlayers as confined reaction environments for transforming simple nitrogen-rich molecules into integrated graphite/2D-material composites, providing a scalable platform for exploring solid-state chemistry and hybrid material synthesis between graphene layers.

## 1. Introduction

The rational construction of graphene-based nanocomposites increasingly relies on molecular-level control over interfacial organization [1]. Graphene nanocomposites represent, at the extreme structural limit, vertically stacked heterostructures composed of atomically thin layers, where interfacial coupling defines the resulting electronic, catalytic, or optical functionality [2,3,4]. High-quality graphene and many related two-dimensional materials are largely insoluble and non-sublimable [5], which limits the applicability of conventional solution-phase or vapor-phase composite fabrication methods commonly used for polymer-based systems [1,6]. As a result, precisely controlled graphene heterostructures are often obtained through labor-intensive manual stacking or mechanical transfer of individual layers, restricting scalability and structural uniformity [7,8]. Developing alternative approaches that enable controlled interface formation through supramolecular organization of precursors and subsequent chemical transformations appears very promising and attractive [9,10,11].

Intercalation chemistry provides a potential pathway toward such control [12]. Graphite possesses a well-defined layered architecture capable of accommodating small molecules through π–π interactions [13,14] and hydrogen-bonding-assisted insertion [15]. Historically, graphite intercalation has been explored for charge-transfer compounds and exfoliation processes. However, beyond charge storage or exfoliation chemistry, intercalation can be viewed as a molecular pre-organization strategy, in which guest molecules are spatially confined between graphene layers prior to subsequent transformation [13,16,17]. In this context, the interlayer space functions not merely as a host structure but as a confined reaction environment capable of influencing molecular packing and reaction pathways [18,19].

Melamine represents a particularly attractive molecular precursor for such a strategy. Its planar triazine core promotes π-stacking interactions with graphitic carbon, while its peripheral amino groups enable hydrogen bonding and directional assembly [20,21]. Moreover, melamine undergoes well-established stepwise thermal condensation to melem and further to graphitic carbon nitride (g-C_3_N_4_), producing extended conjugated carbon–nitrogen frameworks [22,23]. Conventionally, these condensation reactions occur in the bulk phase, often resulting in extensive stacking and limited dispersion when combined with conductive carbon substrates [24]. Furthermore, melamine, as a representative nitrogen-rich organic monomer with a triazine core and three amino functional groups, has been widely explored as a versatile building unit for covalent organic frameworks (COFs) [25]. Its multiple –NH_2_ groups enable condensation with various carbonyl-containing monomers, such as aldehydes or acyl derivatives, facilitating the construction of ordered porous polymer networks. In COF synthesis, melamine typically participates in framework formation via either Schiff base (imine) condensation or amidation reactions [26]. Among these, Schiff base condensation is the most commonly employed route, wherein the amino groups of melamine react with aromatic aldehydes to form –C=N– linkages, leading to the formation of two- or three-dimensional covalent networks [25,27]. Owing to their relatively mild reaction conditions and high degree of structural tunability, such condensation processes have become a key strategy for constructing nitrogen-enriched COFs [28].

In this work, we propose a mechanochemistry-driven strategy that couples interlayer expansion with confined chemical transformation. Under ball-milling conditions, melamine molecules act as molecular wedges and noncovalent intercalants, weakening interlayer van der Waals interactions while establishing π–π interactions and hydrogen-bonding networks with graphitic layers. This process results in a highly expanded and supramolecularly preorganized precursor system within the confined interlayer space, which serves as a structural foundation for subsequent transformations. Building upon this preorganization, we systematically investigate the structural evolution and transformation behavior of the graphite–melamine system. First, a graphite–melamine precursor (G+MA) is constructed via solvent-free mechanochemical treatment, enabling uniform distribution of molecular species within the graphite framework. Subsequent thermal treatment under an inert atmosphere induces confined condensation reactions, leading to the formation of graphite–melem (G–ME) composite, thereby validating the effectiveness of confined transformation within the interlayer environment.

**Scheme 1:**
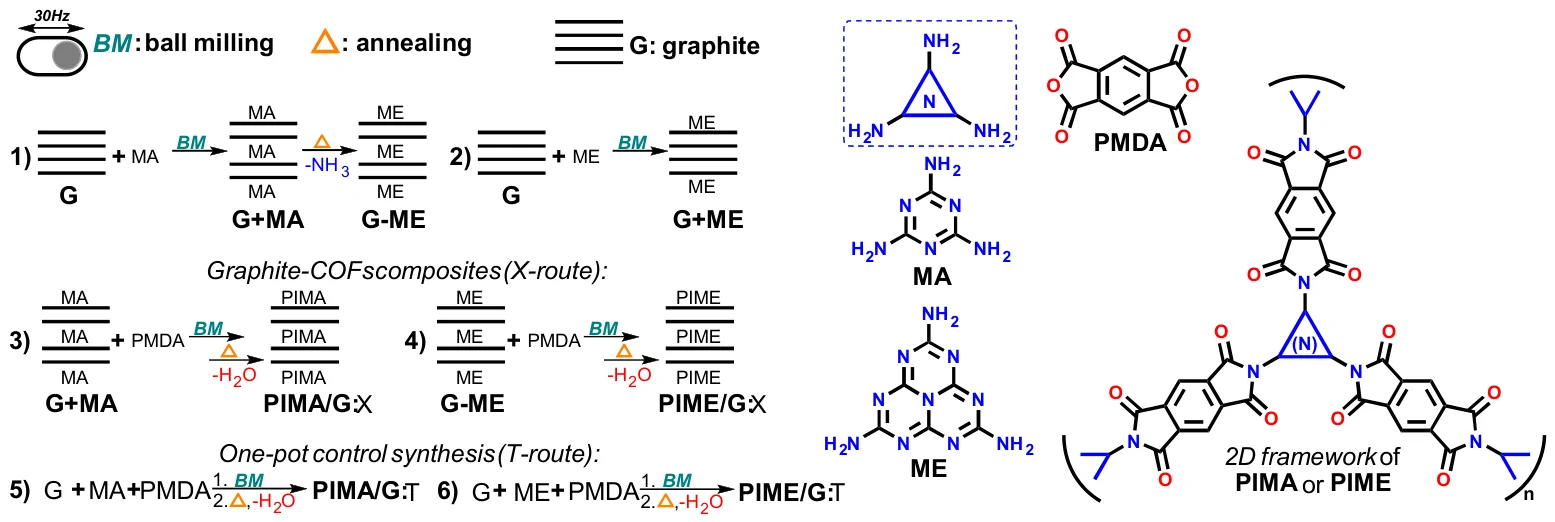
Synthesis and solid-state chemical transformations of graphite–melamine composites.

Furthermore, this strategy is extended to multicomponent systems by introducing external comonomers to enable cooperative polymerization within the confined space. Using melamine (MA) or melem (ME) as nitrogen-containing building units and pyromellitic dianhydride (PMDA) as a complementary monomer, graphite-based composites containing polyimide covalent organic frameworks (PI-COFs) are constructed through solvent-free thermal reactions (Scheme 1).

## 2. Materials and Methods

### 2.1. Materials

Melamine (99.9%) was purchased from Shanghai Dibao Biotechnology Co., Ltd. (Shanghai, China). Graphite powder (99.95%) and pyromellitic dianhydride (PMDA, 99%) were obtained from Aladdin Reagent Co., Ltd. (Shanghai, China). Melem (98%) was purchased from Meryer (Shanghai) Biochemical Technology Co., Ltd. (Shanghai, China). Methanol (HPLC grade) and absolute ethanol (electronic grade, ≥99.8%) were supplied by Yonghua Chemical Co., Ltd. (Changshu, Suzhou, China). All chemicals were used as received without further purification.

### 2.2. Preparation of G+MA and G–ME Precursors

To obtain the graphite–melamine (G+MA) precursor, graphite powder and melamine were subjected to ball milling. The milling conditions were optimized by considering parameters such as milling time, graphite particle size, and mass ratio. The optimization criteria were based on the yield and stability of graphene dispersions after dialysis, reflecting the efficiency of intercalation and exfoliation.

Under optimized conditions, graphite (0.1 g) and melamine (0.3 g) were ball-milled at 30 Hz for 4 h. The resulting G+MA precursor was subsequently thermally treated under a nitrogen atmosphere. The sample was heated to 450 °C at a rate of 3 °C min^−1^ and maintained for 2 h to obtain the graphite–melem (G–ME) composite. After cooling to room temperature, the sample was ground into fine powder using an agate mortar.

### 2.3. Preparation of PI-COFs

PI-COFs were synthesized via solvent-free thermal polymerization using melamine (MA) or melem (ME) with pyromellitic dianhydride (PMDA). For the MA-based system, melamine (0.3 g, 2.38 mmol) was mixed with PMDA at molar ratios of 1:1, 2:3, 4:3, and 1:2. For the ME-based system, melem (0.3 g, 1.37 mmol) was combined with PMDA at molar ratios of 1:1, 1:2, 1:3, 1:4, and 2:1. The mixtures were ball-milled for 30 min to ensure homogeneous dispersion, followed by thermal treatment under nitrogen at 325 °C (5 °C min^−1^) for 5 h. After natural cooling, the products were washed thoroughly with hot water and methanol and dried to obtain fine powders.

### 2.4. Preparation of Graphite/PI-COF Composites

Having identified the optimal conditions for the synthesis of PI-COF, these were extended to the preparation of graphite/PI-COF composites. Graphite/PI-COF composites were prepared based on the preorganization of the nitrogen-rich precursor within graphite. Graphite (0.1 g) and melamine (0.3 g, 2.38 mmol) were used as the base composition, and PMDA was introduced according to the optimized molar ratios described above. Two processing routes were employed.

In the stepwise route (denoted as X), different procedures were employed depending on the precursor system. For the MA-based system, graphite was first ball-milled with melamine (MA) for 4 h, followed by the addition of PMDA and further milling for 1 h. For the ME-based system, graphite was initially ball-milled with MA and subsequently thermally treated to convert MA into melem (ME), forming the intermediate G–ME, after which PMDA was introduced and the mixture was further ball-milled for 1 h.

In the one-pot route (denoted as T), graphite, MA or ME, and PMDA were mixed simultaneously and ball-milled for 5 h prior to thermal treatment.

The subsequent heat treatment and purification procedures were identical to those used for the pure PI-COF samples.

### 2.5. Characterization

Microstructural analysis was carried out using a transmission electron microscope operated at 200 kV (Talos 200X, Thermo Fisher Scientific, Eindhoven, The Netherlands). For TEM characterization, approximately 0.1–1 mg of the powder sample was dispersed in 1–2 mL of absolute ethanol. The resulting suspension was subjected to ultrasonication for 10 min to eliminate particle agglomeration and achieve uniform dispersion. A drop (5–10 μL) of the suspension was then deposited onto a carbon-coated copper grid using a micropipette. After solvent evaporation under ambient conditions, the prepared grid was used for TEM observation.

The morphology of the synthesized composites was examined by a field emission scanning electron microscope (FESEM SU8230, Hitachi Scientific Instruments, Ltd., Tokyo, Japan). Powder XRD analysis was performed using a PANalytical X’Pert3 MRD XL system (Malvern Panalytical, Almelo, The Netherlands) with Ni-filtered Cu Kα radiation at 40 kV and 40 mA, scanning 2θ from 5° to 90° at 2°/min. Raman spectra were acquired on a Horiba HR800 confocal micro-Raman spectrometer (Jobin Yvon, Longjumeau, France) using a 633 nm He-Ne laser with a power output of 17 mW at the sample surface. For statistical Raman analysis, the ID/IG ratio was measured at eleven spatially independent regions for each sample and is reported as the mean ± standard deviation. Fourier transform infrared (FTIR) spectra were recorded on a Bruker VERTEX 70 FTIR spectrometer (Bruker Optics GmbH & Co. KG, Ettlingen, Germany) equipped with a liquid nitrogen-cooled MCT detector. X-ray photoelectron spectroscopy (XPS) analysis was performed using a Thermo Scientific ESCALAB 250Xi system (Thermo Fisher Scientific, East Grinstead, UK) equipped with a monochromatic Al Kα source (hν = 1486.6 eV).

## 3. Results and Discussion

Direct structural characterization of heterogeneous nanocomposites is often challenging because no single technique can unambiguously resolve the spatial arrangement, chemical state, and interfacial organization of all components. A more reliable strategy is therefore to combine complementary characterization methods with carefully selected control experiments, each addressing a specific part of the proposed synthesis concept. In the present study, these experiments were designed to establish the successive premises required for preparing melamine- and melem-derived polyimide-linked materials from intercalated melamine. First, we verified that the ball-milling procedure used here produces a graphite–melamine precursor in which melamine effectively penetrates and separates the graphite layers. Second, we determined whether intercalated melamine retains the characteristic reactivity of bulk melamine by examining its controlled thermal condensation to melem. Demonstrating both effective intercalation and preservation of the known melamine reaction pathway provides the experimental foundation for using intercalated melamine as a chemically active precursor rather than merely as a passive guest species. We then optimized the corresponding melamine- and melem-derived polyimide systems in the absence of graphite to establish suitable reference compositions and structural benchmarks. Finally, these optimized reactions are transferred to the graphite-based precursors to assess whether the corresponding polyimide-forming reactions can proceed from preorganized melamine or melem in close association with the graphene layers.

### 3.1. Mechanochemical Preparation and Controlled Thermal Transformation of the Graphite–Melamine Precursor

Melamine-assisted intercalation [21] and exfoliation [6,20] of graphite, as well as the thermal conversion of graphite–melamine precursors into graphite/g-C_3_N_4_ nanocomposites [14,22], have previously been demonstrated. Together, these studies provide a strong basis for exploring the growth of two-dimensional materials within the confined spaces between graphene layers. However, before applying this concept to the present system, it was necessary to verify that our ball-milling procedure produces effective melamine intercalation and induces graphite exfoliation down to the few-layer regime [29]. After removal of melamine from the ball-milled graphite–melamine precursor, electron-transparent few-layer graphene flakes were obtained, as shown by SEM and TEM in Figure S1.

We then examined whether intercalated melamine retains the characteristic thermal reactivity of bulk melamine. By using a lower annealing temperature of 450 °C, rather than the approximately 550 °C typically employed for the formation of graphite/g-C_3_N_4_ composites [22], the condensation reaction could be selectively arrested at the melem stage. This treatment yielded a graphite–melem composite, denoted G–ME. The structural evolution of this precursor was examined by comparing pristine graphite (G), ball-milled graphite, the graphite–melamine precursor (G+MA), the thermally transformed product (G–ME), and a physical control prepared by directly ball milling graphite with preformed melem (G+ME).

**Figure 1:**
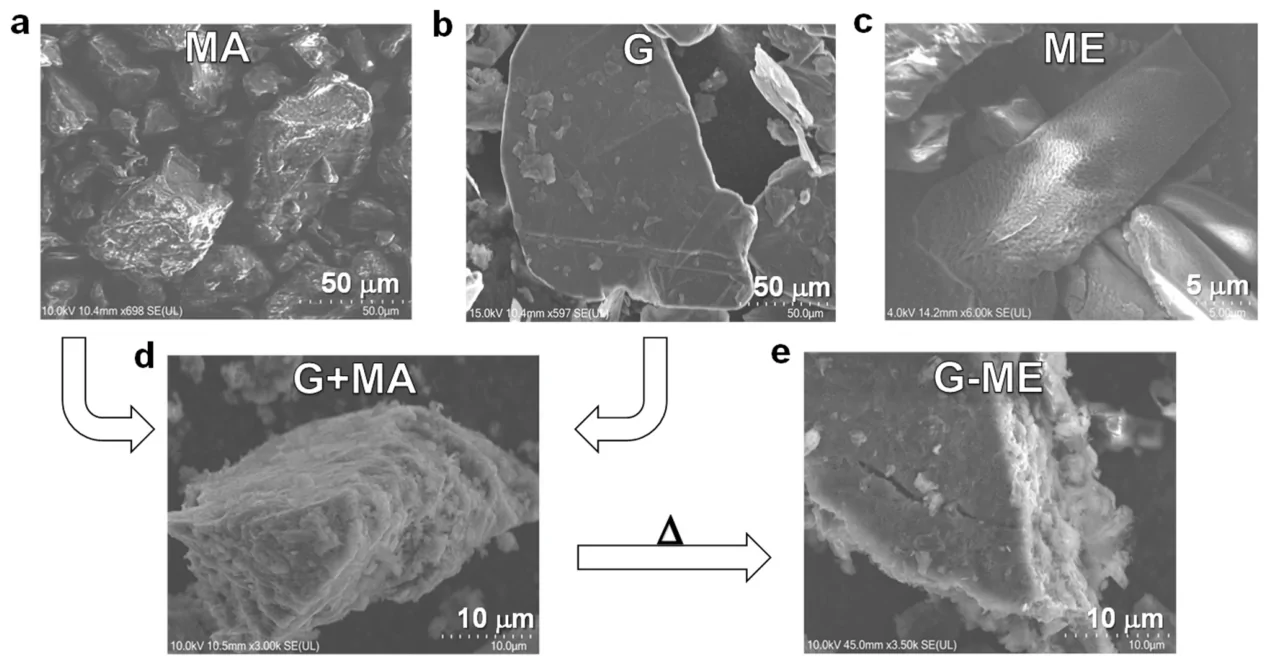
Representative SEM images of (**a**) melamine, MA; (**b**) graphite powder, G; (**c**) melem, ME; (**d**) graphite–melamine, G+MA; and (**e**) graphite–melem, G–ME.

SEM was first used to examine the morphological changes accompanying ball milling and thermal treatment (Figure 1). Pristine melamine consists of irregular block-like particles (average size: 28.8 ± 11.6 µm), whereas preformed melem forms smaller (average size: 4.2 ± 1.4 µm) textured aggregates. Pristine graphite exhibits the expected densely stacked lamellar particles (average size: 107.6 ± 55.4 µm) with smooth basal surfaces and well-defined edges. After ball milling with melamine, the G+MA sample shows substantial fragmentation (average size: 26.2 ± 10.0 µm for G+MA) of the original graphite particles, disruption of the compact lamellar morphology, increased surface roughness, and locally wrinkled or partially delaminated features. These observations are consistent with strong mechanical perturbation and separation of the graphite layers during preparation of the precursor, although SEM alone cannot determine the location of melamine within individual particles. Following thermal treatment, the G–ME sample retains a layered graphite-derived morphology (average size: 14.4 ± 6.1 µm). The broad size distributions reflect the intrinsic polydispersity of the milled and thermally treated powders. Overall, the SEM results show that both mechanochemical processing and subsequent annealing modify the graphite particle morphology, but they do not by themselves distinguish between interlayer incorporation, external deposition, or intimate mixing of the nitrogen-rich phase.

**Figure 2:**
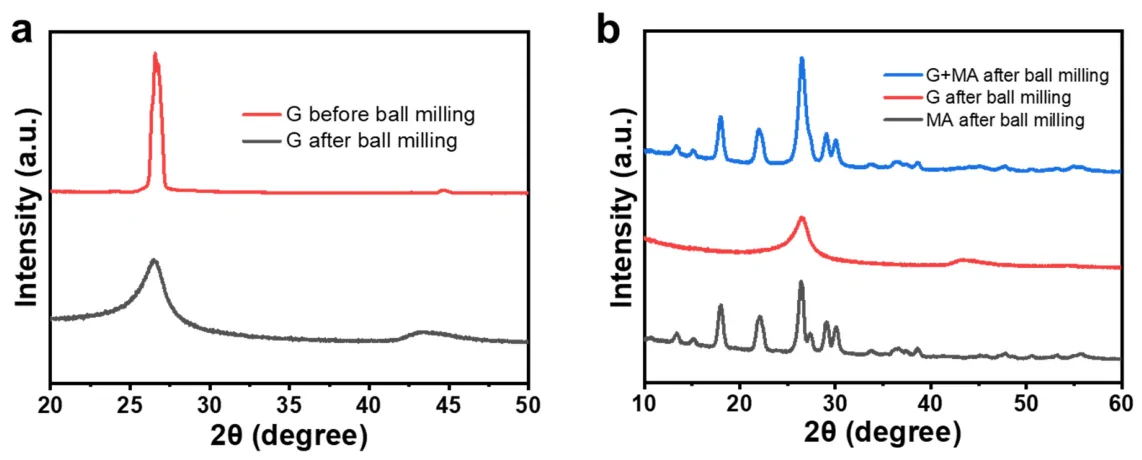
Powder X-ray diffraction (PXRD) patterns of different samples: (**a**) comparison of graphite (G) before and after ball milling; (**b**) ball-milled graphite (G), ball-milled melamine (MA), and the G+MA composite.

Powder X-ray diffraction was next used to evaluate the crystalline phases and changes in graphite ordering. Pristine graphite exhibits a sharp (002) reflection at approximately 26.5°, with a full width at half maximum of 0.21°, consistent with highly ordered layer stacking (Figure 2a). After ball milling, the intensity of this reflection decreases and its FWHM increases to 0.74°, demonstrating a substantial reduction in the coherence of the graphite stacking. This broadening is attributable to the combined effects of particle-size reduction, mechanical strain, defects, and partial loss of long-range order introduced during milling.

The diffraction pattern of the G+MA precursor contains reflections from both graphite and melamine (Figure 2b). The melamine reference used for this comparison was subjected to the same ball-milling treatment as the G+MA sample. Most characteristic melamine reflections remain detectable in G+MA, showing that melamine retains significant crystalline order after mechanochemical treatment. However, the melamine reflections near 26.5° and 27.3° overlap with the graphite (002) region and merge into a broader feature centered at approximately 26.8°. Together with the decreased sharpness of several reflections, this indicates reduced long-range order and improved dispersion of melamine within the mechanically processed mixture. These changes are compatible with the formation of a preorganized graphite–melamine precursor [20].

**Figure 3:**
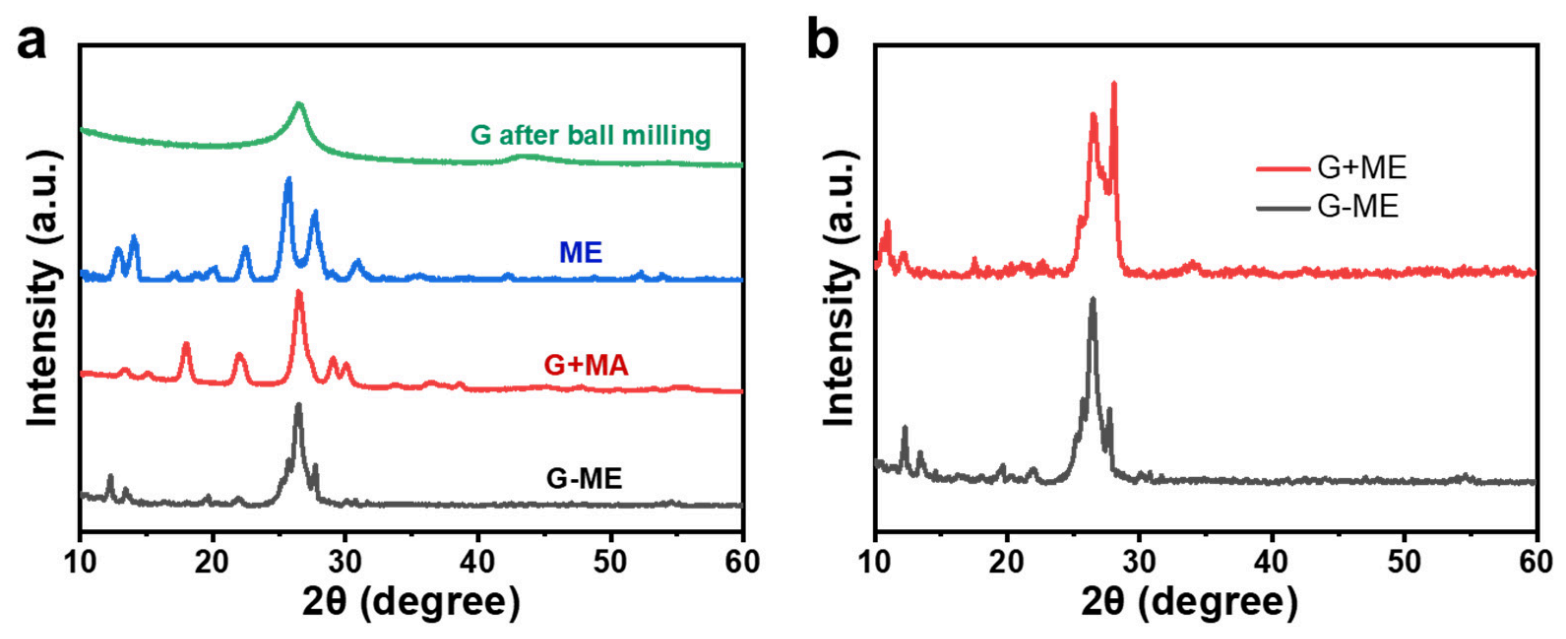
Powder X-ray diffraction (PXRD) patterns of the samples. (**a**) ball-milled graphite (G after ball milling), melem (ME), G+MA composite and G–ME composite; (**b**) comparison of the thermally derived G–ME composite and the ball-milled G+ME sample.

After annealing, the characteristic reflections of melamine disappear, and new diffraction features emerge that resemble those of the melem reference (Figure 3a) and the ball-milled G+ME control sample (Figure 3b). This indicates essentially complete conversion of melamine into a melem-like phase at 450 °C. The graphite (002) reflection remains present and merges with the peaks of melem in the 26–28° region. The complete or partial attenuation of some melem reflections in G–ME and ball-milled G+ME may arise from preferred orientation or altered stacking of the ME-derived layers in association with the graphitic sheets; however, the exact structural arrangement of the ME-derived phase could not be determined from the present XRD data.

**Figure 4:**
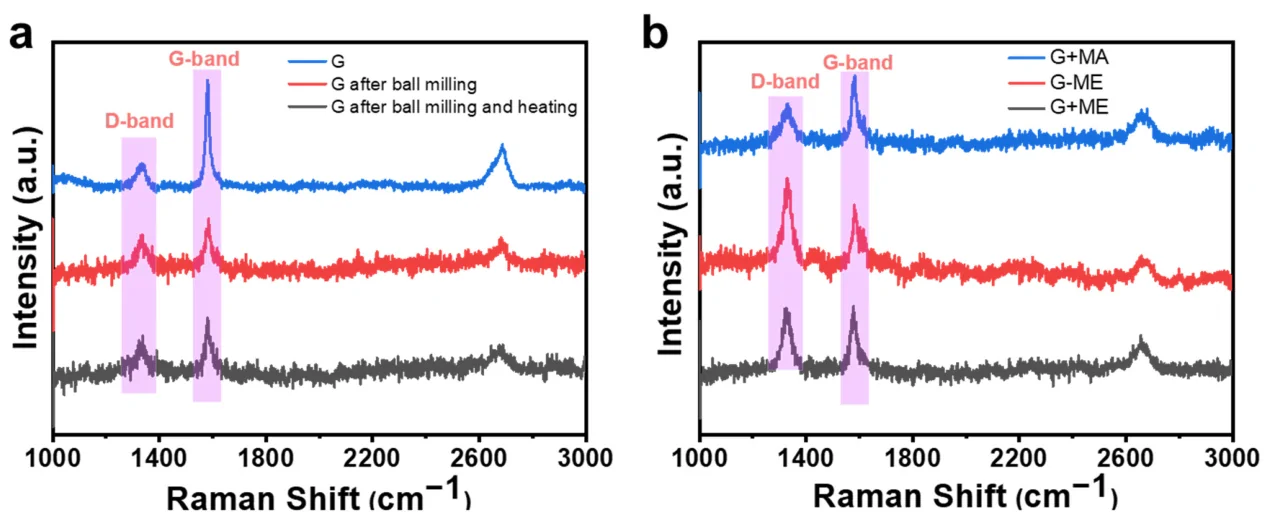
(**a**) Raman spectra of pristine graphite (G), ball-milled graphite (G after ball milling), and graphite after ball milling followed by thermal treatment (G after ball milling and heating); (**b**) Raman spectra of G+MA, G+ME, and G–ME samples.

Raman spectroscopy was useful to assess the changes in the graphitic carbon framework during milling and thermal treatment (Figure 4). Pristine graphite exhibits a weak D band and a sharp G band, with an average I_D_/I_G_ ratio of 0.176 ± 0.082. After ball milling, this value increases to 0.577 ± 0.097, confirming that mechanical treatment introduces substantial disorder into the graphitic structure. Heating the ball-milled graphite control gives an I_D_/I_G_ value of 0.501 ± 0.171. The slight decrease relative to ball-milled graphite may reflect limited rearrangement of some mechanically generated defects during annealing, although the carbon framework remains considerably more disordered than pristine graphite. The graphite–melamine precursor exhibits a higher average I_D_/I_G_ ratio of 0.979 ± 0.178, indicating that milling graphite in the presence of melamine produces a carbon structural state different from milling graphite alone. After thermal conversion, the G–ME sample gives an I_D_/I_G_ ratio of 1.407 ± 0.110, while the physical G+ME mixture gives 1.316 ± 0.139. The relatively close values indicate that both the presence of melem-derived material and the applied processing conditions substantially affect the Raman response of the carbon framework.

**Figure 5:**
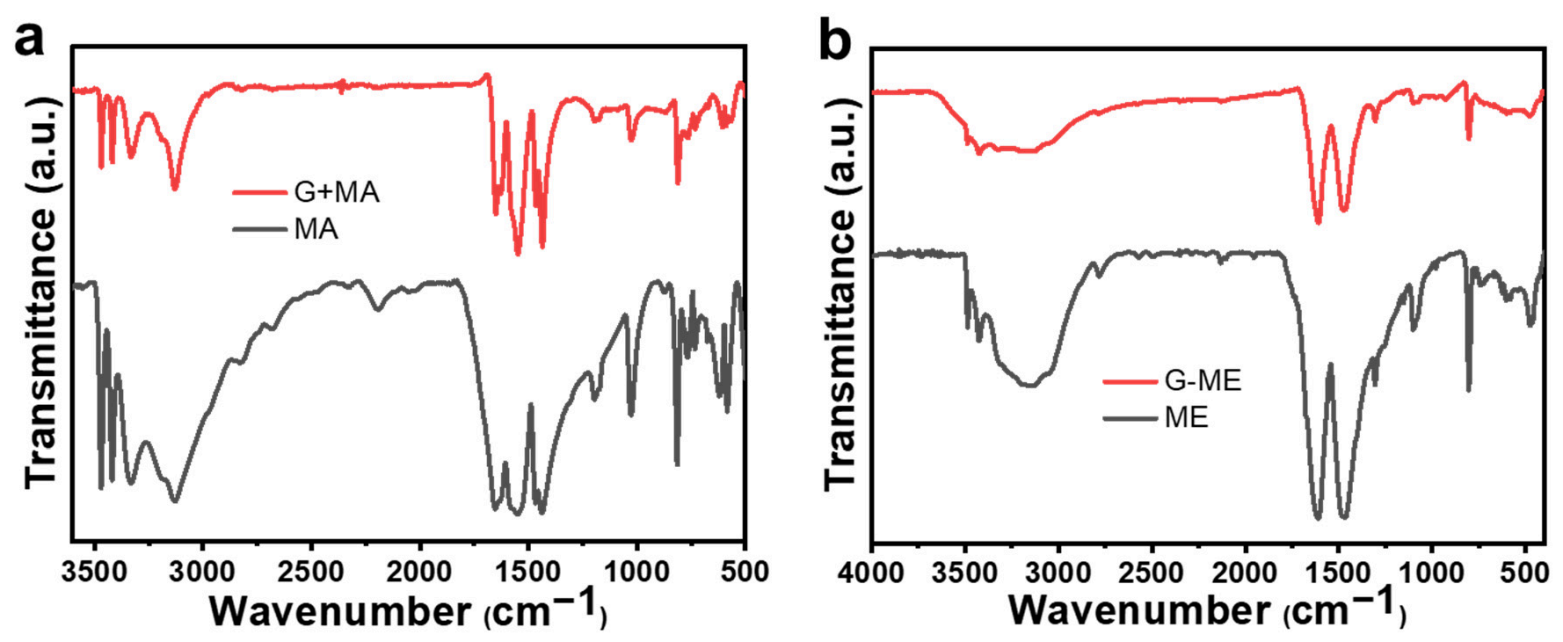
Fourier transform infrared (FTIR) spectra of different samples. (**a**) Melamine (MA) and the G+MA composite precursor; (**b**) melem (ME) and the G–ME composite material.

FTIR spectroscopy was used to follow the chemical conversion of melamine during annealing (Figure 5). Melamine exhibits broad N–H stretching bands between approximately 3100 and 3500 cm^−1^ and multiple C–N and C=N vibrations in the 1200–1700 cm^−1^ region. Following thermal treatment, the G–ME spectrum changes substantially: the N–H stretching bands decrease in intensity, while bands associated with condensed conjugated C–N and C=N structures become more prominent. The resulting spectrum closely resembles that of the melem reference and is consistent with condensation of melamine to a melem-like product in G–ME.

**Figure 6:**
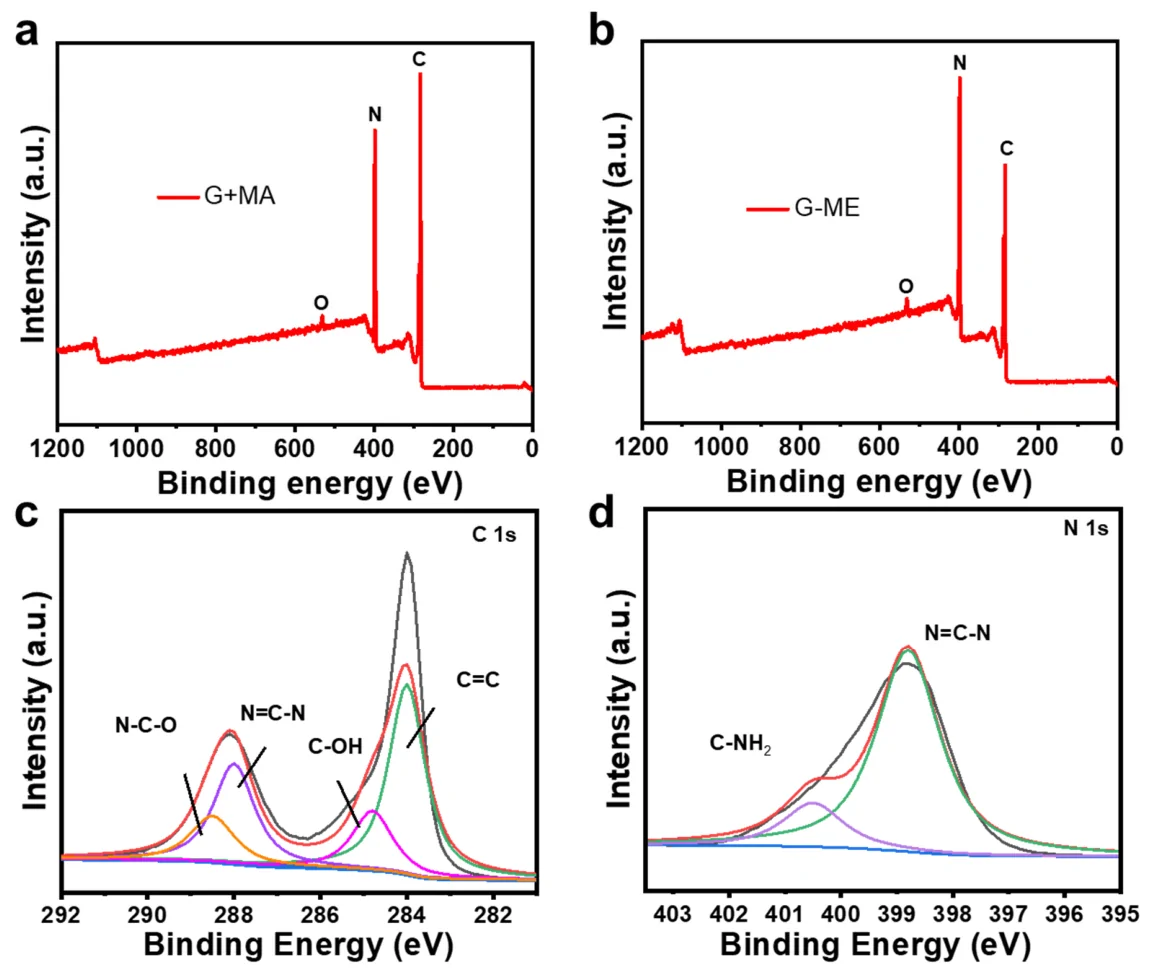
X-ray photoelectron spectroscopy (XPS) analysis of samples at different stages. (**a**,**b**) Survey spectra of (**a**) the G+MA precursor composite and (**b**) the G–ME composite. (**c**,**d**) High-resolution XPS spectra of the G–ME sample: (**c**) C 1s spectrum and (**d**) N 1s spectrum.

XPS further confirms the coexistence of graphitic carbon and condensed nitrogen-containing species in the thermally transformed composite (Figure 6). The survey spectra of G+MA and G–ME contain strong C 1s and N 1s signals together with a weaker O 1s contribution (the latter most likely arising from surface oxidation of graphite during ball milling). The measured surface nitrogen content increases upon condensation of melamine to melem (from an estimated 29.18 at.% in G+MA to 35.36 at.% in G–ME). The high-resolution C 1s spectrum of G–ME contains components attributable to graphitic C–C/C=C, C–N, and N–C=N or C=N environments, while the N 1s spectrum includes contributions assigned to conjugated C=N–C nitrogen together with C–N and residual amino-related nitrogen. These chemical states are consistent with the formation of a condensed melem-like structure that retains terminal amino groups, as expected for a reaction stopped at the melem stage rather than continued toward more extensively condensed g-C_3_N_4_.

Taken together, the SEM, PXRD, Raman, FTIR, and XPS results establish that the mechanochemically prepared graphite–melamine precursor undergoes a controllable thermal transformation under the selected annealing conditions. Melamine is converted into a melem-like phase while the graphite framework remains present. These results also demonstrate that intercalated MA in G+MA can undergo the same thermal transformation as bulk MA.

### 3.2. Establishing and Optimizing Melamine- and Melem-Derived Polyimide Reference Systems

Before transferring the polyimide-forming reaction to graphite-based precursors, it was necessary to establish suitable reference systems in the absence of graphite. This step serves two purposes. First, it confirms that melamine and melem can react with pyromellitic dianhydride (PMDA) under the solvent-free conditions employed in this study to form polyimide-linked materials. Second, it identifies the precursor ratios that provide the most complete conversion and the highest degree of structural order. These optimized products then serve as reference materials for evaluating whether analogous reactions can proceed from melamine or melem preorganized within graphite.

**Figure 7:**
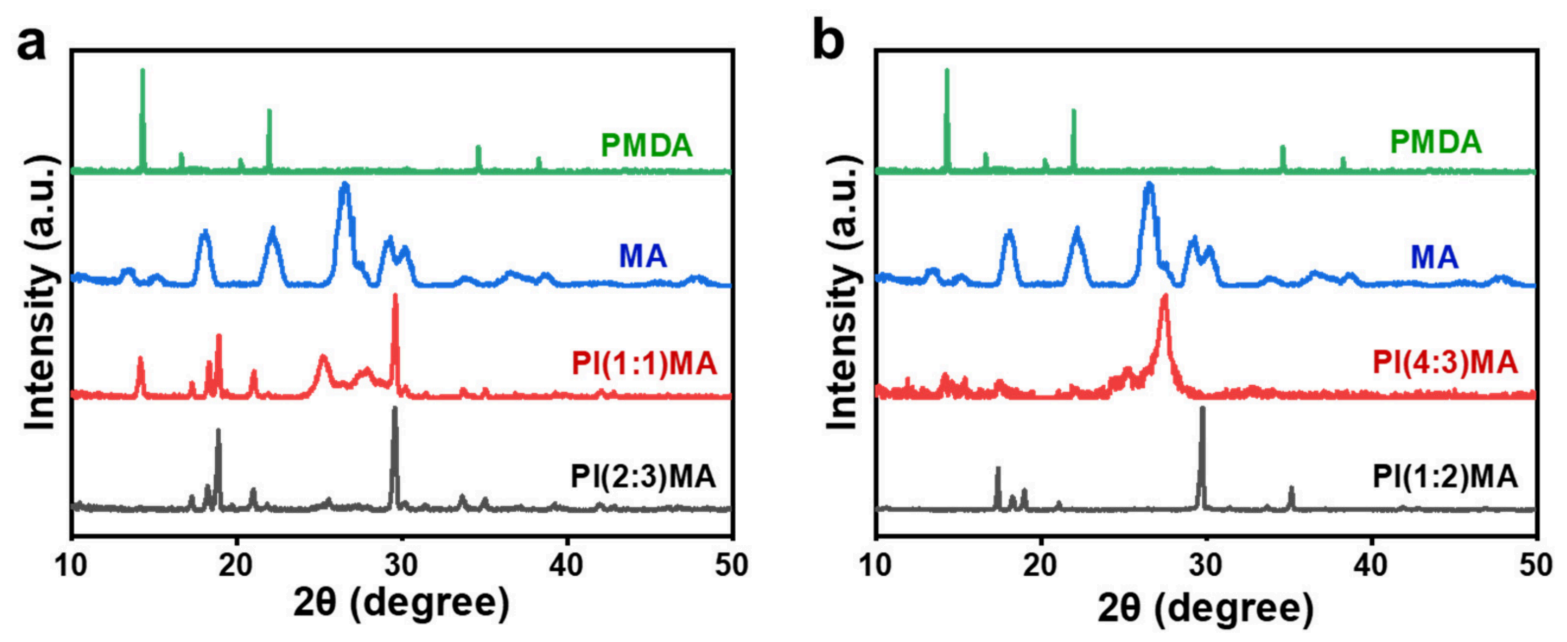
X-ray diffraction (XRD) patterns of PI-COFMA samples prepared with different MA:PMDA molar ratios. (**a**) Comparison of diffraction features of PI(1:1)MA and PI(2:3)MA samples with those of the monomers PMDA and MA; (**b**) XRD patterns of samples synthesized under monomer-excess conditions, including MA-excess PI(4:3)MA and PMDA-excess PI(1:2)MA.

The melamine-based series was first examined by powder X-ray diffraction (Figure 7). The diffraction patterns depend strongly on the MA:PMDA ratio, indicating that precursor stoichiometry governs both phase development and long-range structural order. Because the idealized two-dimensional hexagonal covalent structure shown in Scheme 1 requires an MA:PMDA ratio of 2:3, the PI(2:3)MA sample was compared with compositions containing either less PMDA, namely PI(1:1)MA and PI(4:3)MA, or excess PMDA, namely PI(1:2)MA.

All reflections observed for PI(2:3)MA are also present in the PI(1:1)MA pattern, suggesting that the same principal phase forms in both samples. However, PI(1:1)MA also exhibits an additional reflection at 14.3° and broad features in the 25–29° region, indicating the presence of additional phases, likely enriched in melamine. Increasing the melamine content further has a much stronger effect. The PI(4:3)MA sample displays only a few broad reflections, and the characteristic diffraction features shared by PI(2:3)MA and PI(1:1)MA are no longer evident, indicating a substantial loss of structural order and the formation of a structurally different and more disordered product.

By contrast, increasing the PMDA content beyond the ideal stoichiometric ratio has a less pronounced effect. The PI(1:2)MA sample remains crystalline and retains clear structural similarity to PI(2:3)MA, although fewer reflections are observed. Overall, the XRD results indicate that excess melamine disrupts formation of the ordered PI(2:3)MA-related phase more strongly than excess PMDA.

**Figure 8:**
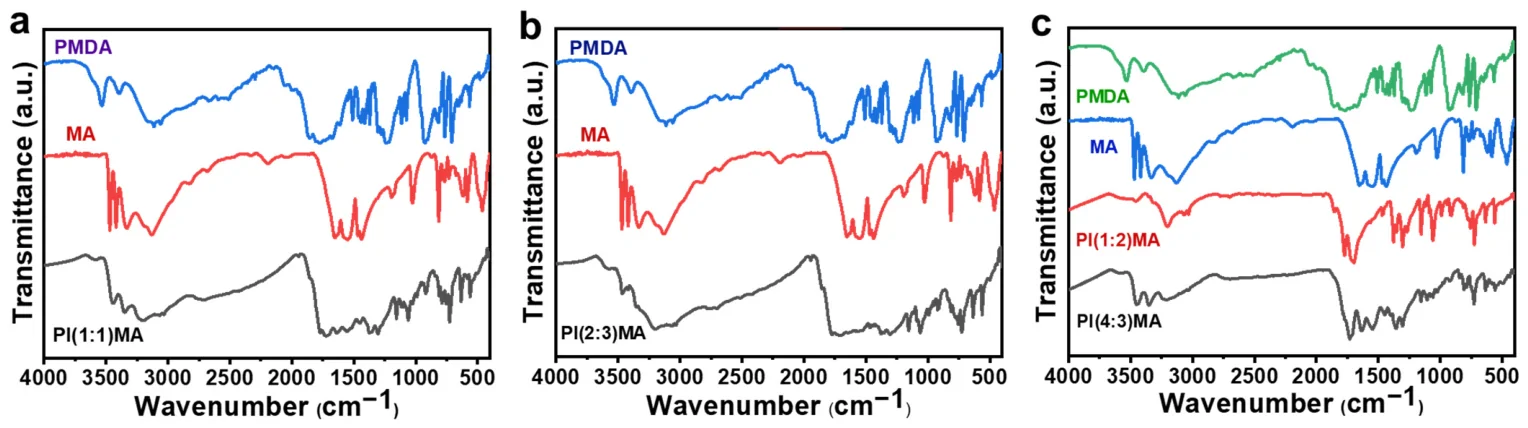
Fourier transform infrared (FTIR) spectra of PI-COFMA samples prepared with different MA:PMDA molar ratios. (**a**) FTIR spectra of MA, PMDA, and PI(1:1)MA; (**b**) FTIR spectra of MA, PMDA, and PI(2:3)MA; (**c**) FTIR spectra of MA, PMDA, PI(1:2)MA, and PI(4:3)MA. For clarity and convenience, the individual FTIR spectra of the full MA-based series are also provided in Figure S2.

FTIR spectroscopy confirms that the diffraction changes are accompanied by imide formation (Figure 8). All melamine-derived products exhibit characteristic bands near 1772, 1725, 1376, and 725 cm^−1^, assigned to imide-related vibrations. The presence of these bands demonstrates that the reaction between the amino groups of melamine and the anhydride groups of PMDA occurs under the applied solid-state conditions.

Nevertheless, the extent of conversion varies with precursor ratio. In PI(1:1)MA, a strong and broad N–H stretching envelope remains between approximately 3120 and 3470 cm^−1^, indicating substantial residual amino functionality. The imide-related bands are also comparatively broad, consistent with a chemically and structurally heterogeneous product. In PI(2:3)MA, the N–H stretching bands are markedly reduced, the anhydride-related band near 1850 cm^−1^ is absent, and the imide bands become sharper and better defined. These changes indicate more complete consumption of the starting functional groups and a more uniform imide-containing structure.

The off-stoichiometric compositions show the expected signatures of incomplete conversion. In the PMDA-rich PI(1:2)MA sample, the N–H signal is strongly suppressed, but weak residual anhydride-related features remain. Conversely, the melamine-rich PI(4:3)MA sample retains pronounced N–H absorption, consistent with unreacted or terminal amino groups. In both cases, the imide-related bands are broader or less well resolved than in PI(2:3)MA. The FTIR data therefore support the XRD-based conclusion that the 2:3 composition gives the most complete and structurally regular melamine-derived polyimide-linked product.

**Figure 9:**
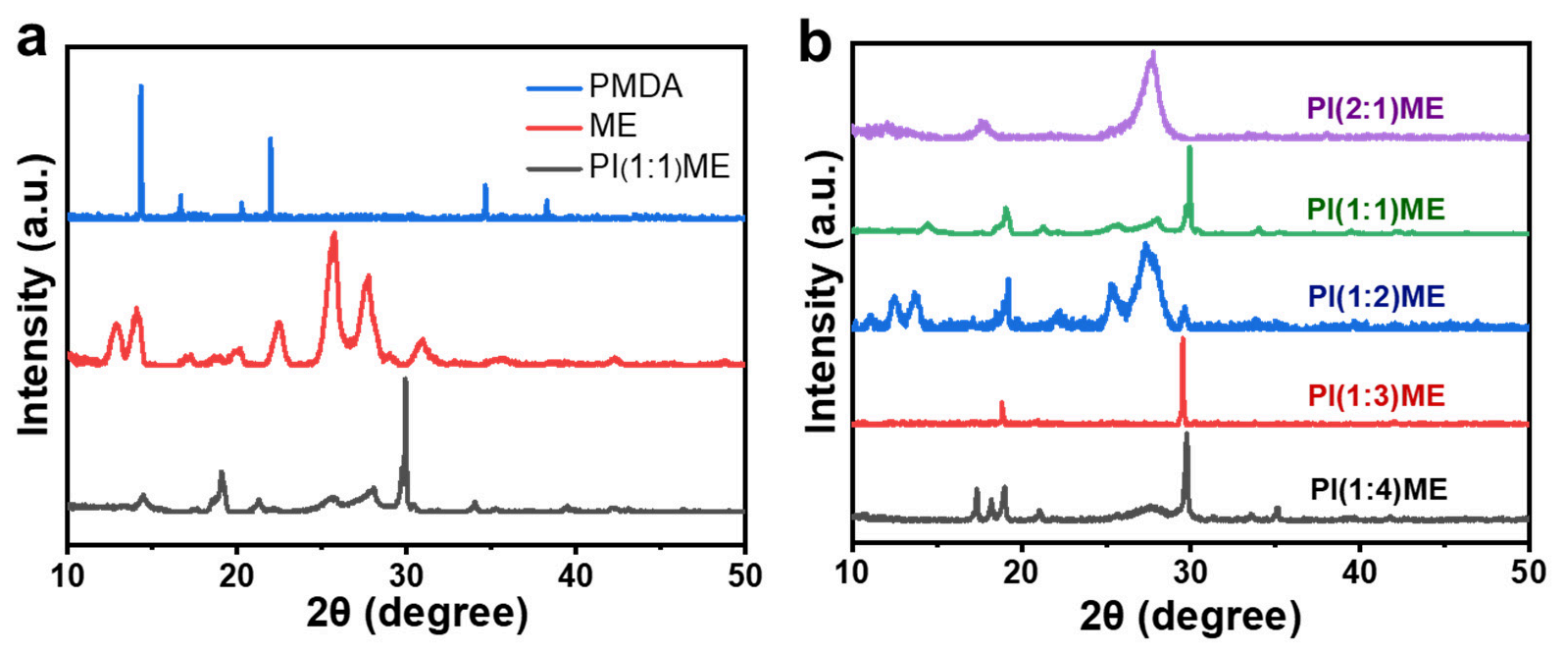
X-ray diffraction (XRD) patterns of PI-COFME samples prepared with different ME:PMDA molar ratios. (**a**) Comparison of the diffraction features of PI(1:1)ME with those of the monomers PMDA and ME; (**b**) XRD patterns of PI-COFME samples synthesized at varying ME:PMDA ratios, including PI(2:1)ME, PI(1:1)ME, PI(1:2)ME, PI(1:3)ME, and PI(1:4)ME.

The corresponding melem-derived series exhibits a stronger, although less straightforward, dependence on precursor stoichiometry (Figure 9). PI(1:3)ME displays the sharpest diffraction features, including distinct reflections at 18.9° and 29.6°. Samples containing lower proportions of PMDA exhibit additional broad features, likely associated with partially reacted melem-derived polyimide species, whereas PI(1:4)ME forms a crystalline product containing additional reflections not observed in the other samples. These results reveal a relatively narrow compositional window for the formation of an ordered melem-derived polyimide-linked phase. This greater sensitivity to stoichiometry may arise from the larger size, lower mobility, and more rigid hydrogen-bonding and π-stacking interactions of melem relative to melamine. Under solvent-free conditions, these factors are expected to restrict molecular rearrangement and make efficient development of the polyimide-linked structure more dependent on the local precursor ratio. On this basis, PI(1:3)ME was selected as the reference composition for the subsequent graphite-composite experiments.

**Figure 10:**
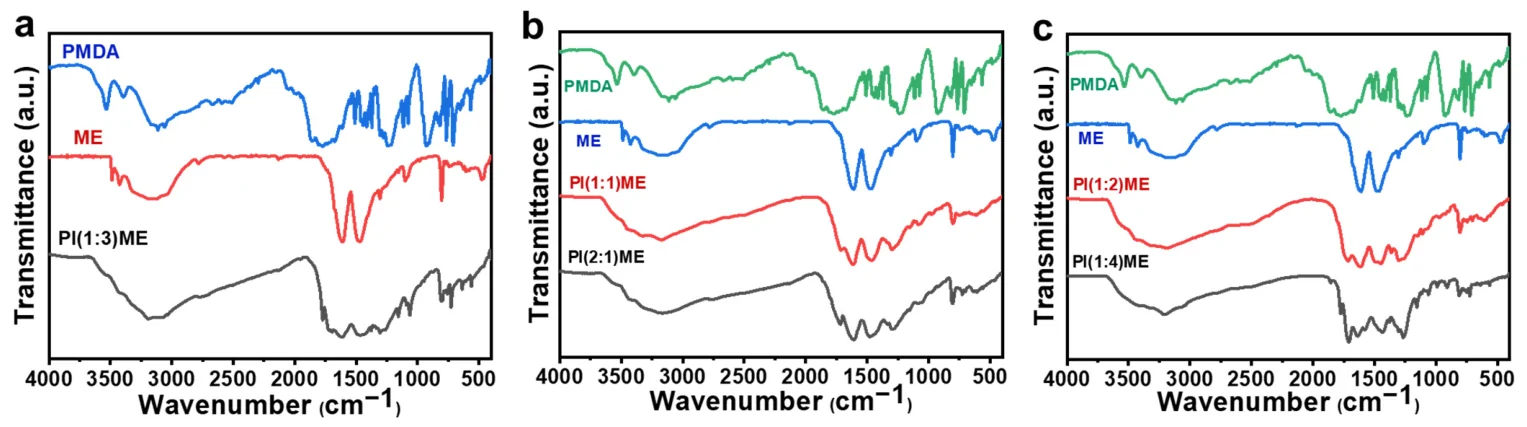
FTIR spectra of samples prepared with different ME:PMDA ratios. (**a**) FTIR spectra of ME, PMDA, and the optimized composition PI(1:3)ME; (**b**) FTIR spectra of samples under ME-excess conditions, including PI(1:1)ME and PI(2:1)ME; (**c**) FTIR spectra of samples with varying PMDA content, including PI(1:2)ME and PI(1:4)ME. For clarity and convenience, the individual FTIR spectra of the full ME-based series are also provided in Figure S3.

FTIR spectroscopy provides independent evidence for the chemical conversion of the melem-based series (Figure 10). The PI(1:3)ME sample exhibits the characteristic imide-related bands near 1772, 1725, 1376, and 725 cm^−1^. At the same time, the N–H stretching bands between 3100 and 3500 cm^−1^ are strongly reduced, and the anhydride-related signal near 1850 cm^−1^ is no longer detected. These features indicate substantial consumption of both amino and anhydride groups and are consistent with extensive imide formation.

The off-optimal compositions show evidence of less complete reaction. In the melem-rich samples, bands near 1610 and 1460 cm^−1^ remain prominent and are accompanied by weaker imide signals, indicating retention of melem-related structural motifs and incomplete conversion. An additional feature near 1710 cm^−1^ becomes more pronounced as the melem content increases, suggesting the presence of partially reacted carbonyl-containing species. In the PMDA-rich compositions, residual anhydride-related absorption becomes detectable, particularly for PI(1:4)ME, while the imide bands are less well defined than in PI(1:3)ME. The individual spectra of the melem-based series are provided in Figure S3.

Taken together, the XRD and FTIR results establish that both melamine and melem undergo solvent-free reaction with PMDA to form polyimide-linked products, but that the degree of conversion and structural order depends strongly on precursor stoichiometry. PI(2:3)MA and PI(1:3)ME provide the clearest combination of imide formation, suppression of residual precursor signatures, and development of well-defined diffraction features. These two compositions were therefore selected as the principal reference systems for determining whether analogous polyimide-forming reactions can proceed when melamine or melem is preorganized within graphite.

### 3.3. Formation of Graphite/Polyimide-Linked Composites from Preorganized Precursors

Having established suitable melamine- and melem-derived polyimide reference systems, we next examined whether analogous reactions could be transferred to graphite-based precursors. Two synthetic routes were compared to evaluate the influence of precursor preorganization. In the stepwise route, denoted X, melamine or melem was first associated with graphite before PMDA was introduced. In the one-pot route, denoted T, graphite, the nitrogen-rich precursor, and PMDA were combined from the beginning. The comparison was therefore designed to determine whether prior organization of melamine or melem within the graphite matrix affects the structural development and morphology of the resulting composites.

**Figure 11:**
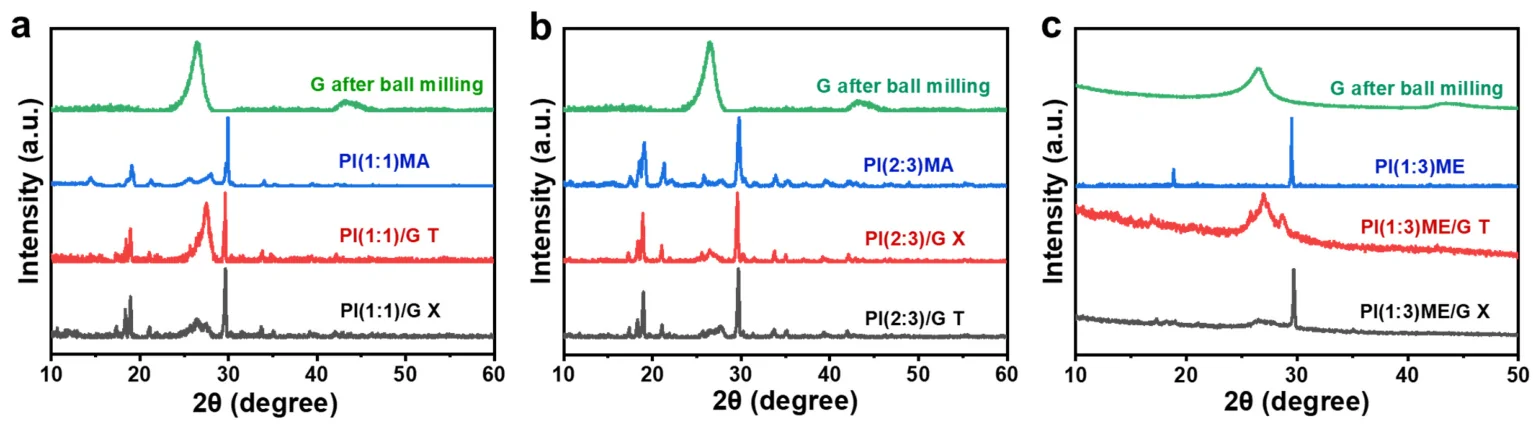
X-ray diffraction (XRD) patterns of graphite/PI composites prepared via different ball-milling routes and monomer ratios. (**a**) Comparison of XRD patterns of ball-milled graphite, PI(1:1)MA, and their corresponding composites PI(1:1)MA/G T and PI(1:1)MA/G X; (**b**) comparison of XRD patterns of ball-milled graphite, PI(2:3)MA, and their corresponding composites PI(2:3)MA/G T and PI(2:3)MA/G X; (**c**) comparison of ball-milled graphite, PI(1:3)ME, and the corresponding composites prepared by the two synthetic pathways.

For the melamine-derived system, both the 1:1 and 2:3 MA:PMDA compositions were investigated (Figure 11a,b). In all cases, the characteristic reflections of the corresponding polyimide-linked reference materials can be readily identified in the graphite-based composites, indicating that the polyimide-forming condensation proceeds in a comparable manner when melamine is associated with graphite. Interestingly, the presence of graphite appears to improve the selectivity and structural order of the product formed from the 1:1 composition, because both PI(1:1)MA/G T and PI(1:1)MA/G X display diffraction features closely resembling those of the reference PI(2:3)MA phase. The XRD patterns of the melamine-derived graphite composites are otherwise quite similar, and only limited differences can be distinguished between the two preparation routes. The most noticeable exception is PI(1:1)MA/G T, which exhibits a more intense broad feature in the graphite-related diffraction region. This may indicate a less homogeneous distribution of the graphite and polyimide-derived components in the one-pot sample, although this interpretation cannot be established from XRD alone. The effect of precursor preorganization is much clearer in the melem-derived system (Figure 11c). The stepwise X route gives a diffraction pattern closely matching that of the reference PI(1:3)ME material, indicating selective conversion to the targeted polyimide-linked phase. In contrast, the one-pot T route does not reproduce the characteristic PI(1:3)ME diffraction pattern and instead yields broader and more complex features, consistent with a less selective and more heterogeneous transformation.

Taken together, the XRD results show that the sequence of precursor addition strongly influences the structural organization of the products. The stepwise route generally gives simpler and better-preserved diffraction patterns, whereas the one-pot route produces broader and more complex features, particularly in the melem-based system. These observations are consistent with a beneficial effect of precursor preorganization, although XRD alone cannot determine whether the polyimide-linked phase is located between graphite layers, at external surfaces, or in closely associated domains.

**Figure 12:**
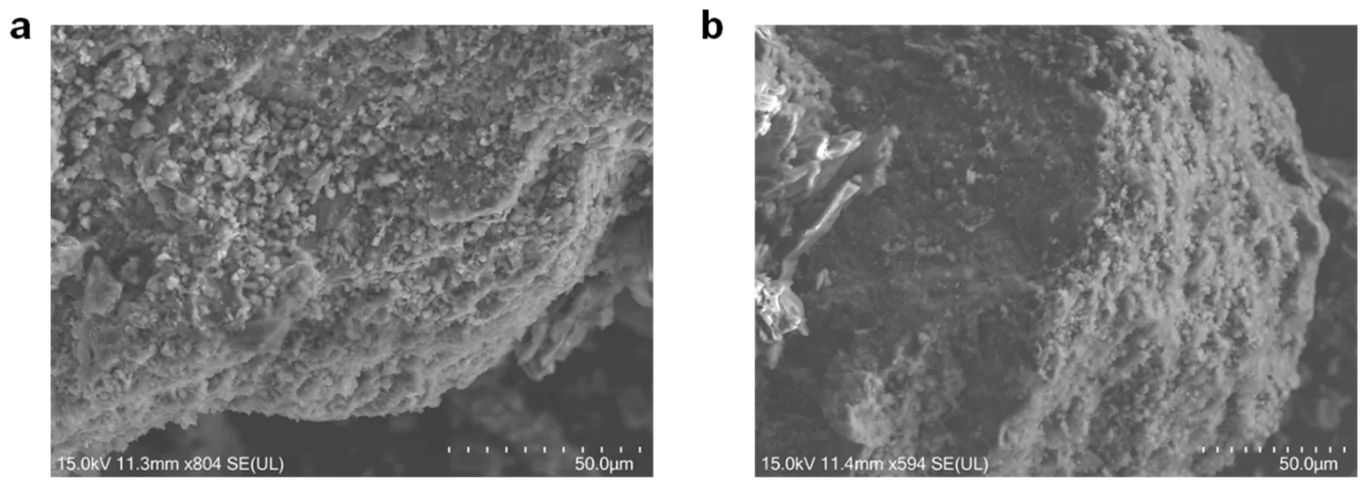
Scanning electron microscopy (SEM) images of PI(2:3)MA/G composites prepared via different pathways: (**a**) PI(2:3)MA/G T (T route) and (**b**) PI(2:3)MA/G X (X route).

Representative composites prepared from the optimized melamine- and melem-derived systems were subsequently examined by SEM. Figure 12 compares PI(2:3)MA/G composites obtained by the one-pot and stepwise routes. Both samples contain a mixture of layered and particulate features. The layered regions are consistent with graphite-derived particles, whereas the smaller particulate domains are likely associated with polyimide-rich material formed outside or at the surfaces of the graphite particles. The one-pot PI(2:3)MA/G T sample displays a relatively heterogeneous morphology consisting of irregular particles and exposed layered regions. The stepwise PI(2:3)MA/G X sample appears somewhat more compact and exhibits a more continuous layered texture. Image analysis gives mean particle sizes of 66.6 ± 14.5 µm for the one-pot sample and 78.2 ± 22.9 µm for the stepwise sample.

**Figure 13:**
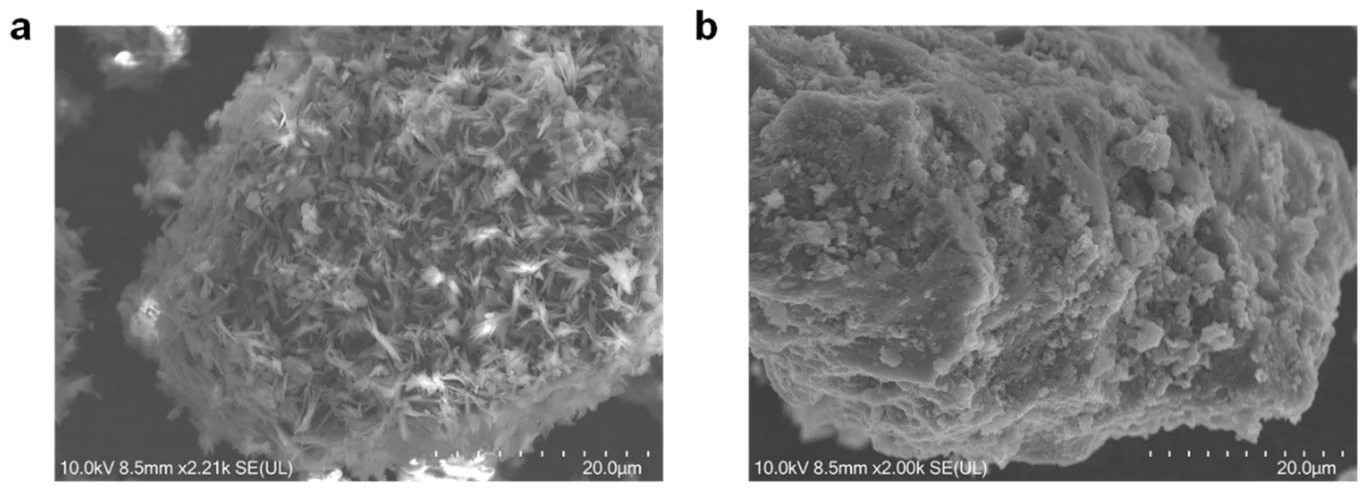
Scanning electron microscopy (SEM) images of PI(1:3)ME/G composites prepared via different pathways: (**a**) PI(1:3)ME/G T (T route) and (**b**) PI(1:3)ME/G X (X route).

A larger morphological difference is observed for the melem-derived composites (Figure 13). The one-pot PI(1:3)ME/G T sample forms highly aggregated particles with rough surfaces and radially spreading sheets. This morphology is consistent with heterogeneous reaction and with the formation of substantial material outside the graphite particles. The stepwise PI(1:3)ME/G X sample is more compact and displays a more continuous layered morphology with smoother surfaces and fewer pronounced radial aggregates, consistent with stronger morphological integration between the graphite-derived and polyimide-derived components.

The SEM results therefore support the conclusion that the sequence of precursor addition affects the macroscopic organization of the composites. In both the melamine- and melem-derived systems, the stepwise route tends to produce more compact and layered morphologies, whereas the one-pot route leads to greater surface roughness and heterogeneity. This difference is particularly pronounced for melem, consistent with the stronger route dependence observed by XRD. Nevertheless, SEM cannot establish the nanoscale location of the polyimide-linked phase and cannot by itself distinguish interlayer formation from surface deposition or intimate particle-level mixing. FTIR spectroscopy was used to determine whether the characteristic imide chemistry identified in the reference materials is retained in the graphite composites. Because the stepwise samples exhibited the most clearly preserved diffraction features and the most integrated morphologies, they were selected for subsequent characterization.

**Figure 14:**
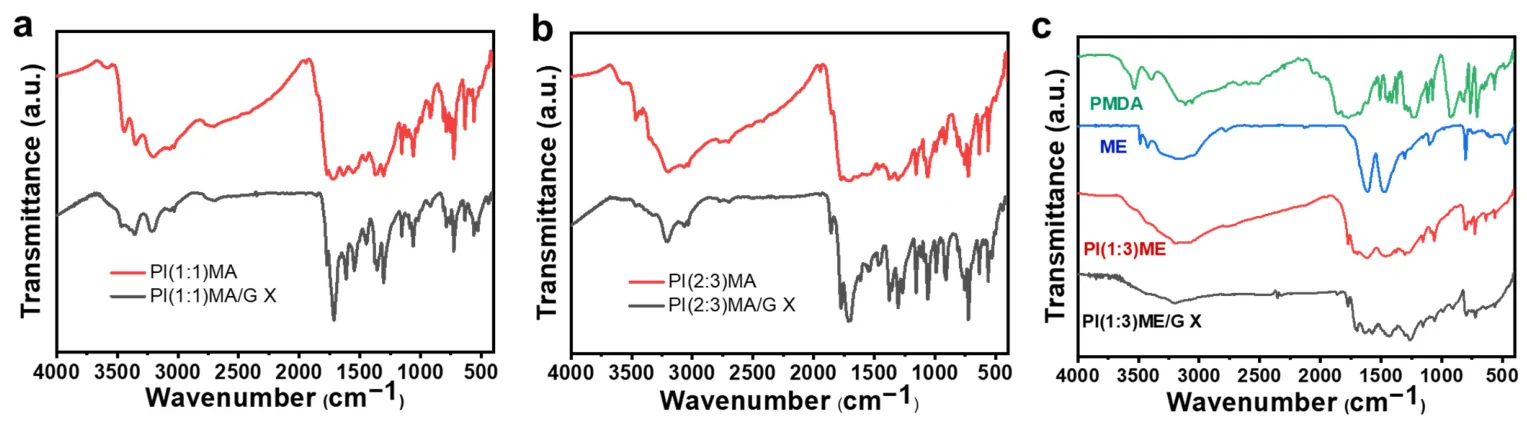
FTIR spectra of PI and their corresponding composites with different compositions. (**a**) FTIR spectra of PI(1:1)MA and PI(1:1)MA/G X; (**b**) FTIR spectra of PI(2:3)MA and PI(2:3)MA/G X; (**c**) FTIR spectra of PMDA, ME, PI(1:3)ME, and the corresponding composite material. For clarity, the individual FTIR spectra of the full MA- and ME-based series are provided in Figures S2 and S3, respectively.

For the melamine-derived systems, both PI(1:1)MA/G X and PI(2:3)MA/G X retain the characteristic imide-related bands of their corresponding graphite-free references in the 1700–1800 cm^−1^ region (Figure 14a,b). In both composites, the N–H stretching envelope at approximately 3300–3500 cm^−1^ is reduced, particularly for the 2:3 composition, indicating a lower relative contribution from residual amino-containing species. Minor differences in peak shape may reflect changes in local packing, orientation, or compositional heterogeneity.

Similarly, the melem-derived PI(1:3)ME/G X composite preserves the diagnostic imide bands of the PI(1:3)ME reference, while the N–H stretching region remains weak (Figure 14c). No new absorption bands appear in any of the composites after addition of graphite. The FTIR results therefore confirm that the characteristic MA–PMDA and ME–PMDA imide-forming reactions proceed in the presence of graphite and that the principal chemical structures of the products are retained. At the same time, the absence of new bands suggests that graphite interacts predominantly through noncovalent association and confinement rather than through formation of new covalent bonds. FTIR does not, however, resolve the spatial distribution of the graphite and polyimide-linked components.

To probe the composites at the nanoscale, TEM, HRTEM, SAED, and STEM–EDX analyses were performed on the optimized PI(2:3)MA/G and PI(1:3)ME/G samples prepared by the stepwise route. The corresponding reference polyimide-linked materials form plate-like particles with well-defined edges, and elemental mapping shows a uniform distribution of carbon, nitrogen, and oxygen across individual particles (Figures S4 and S5).

In the graphite composites, HRTEM reveals well-resolved graphite lattice fringes. In some edge regions, these fringes become less continuous and more dispersed than in pristine graphite, indicating local perturbation of the layer stacking (Figure S6). Analysis of the lattice images gives an average spacing of approximately 0.35 nm, slightly larger than the 0.335 nm spacing of pristine graphite. The distribution is also broader, consistent with local variation in stacking and modest expansion of the layered structure. The corresponding FFT contains periodicities near 0.35 and 0.20 nm, assignable to the graphite (002) interlayer spacing and the in-plane graphitic spacing, respectively (Figure S7).

The approximately 0.35 nm periodicity is close to values expected for both expanded graphitic stacking and interlayer stacking in polyimide-derived layered materials. It therefore cannot be assigned uniquely to either component. No separate lattice periodicity attributable exclusively to the polyimide-linked phase was resolved, most likely because of its lower electron density, limited domain size, and sensitivity to the electron beam. SAED shows discrete spots associated with the crystalline graphitic phase but does not independently resolve a distinct polyimide-derived diffraction pattern.

STEM–EDX mapping reveals substantial compositional heterogeneity within the composites. Some regions are dominated by carbon and contain only weak nitrogen signals, consistent with graphite-rich domains (Figure S8). Other regions contain substantial nitrogen together with carbon within the same particles (Figure S9). Point spectra and domain-resolved analyses show that the C:N ratio varies markedly between different regions, confirming the coexistence of graphite-rich and nitrogen-rich domains (Figure S10).

Importantly, in nitrogen-containing regions, the nitrogen signal is not restricted to the external particle boundary but is distributed throughout the projected particle area. This observation argues against a model in which the polyimide-linked material exists only as a uniform external coating. At the same time, the carbon and nitrogen signals remain largely co-located at the available spatial resolution, and clearly alternating graphite-rich and nitrogen-rich layers are not resolved. The EDX data therefore support intimate association and domain-level mixing of graphite and polyimide-derived components but do not unambiguously establish a regularly alternating interlayer architecture.

Taken together, the XRD, SEM, FTIR, TEM, and EDX results demonstrate that polyimide-linked materials can be formed from the intercalated graphite-based precursors through melamine- and melem-derived reactions. The characteristic imide chemistry and major diffraction features of the optimized reference products are retained in the composites, while the processing route strongly influences their structural and morphological organization. The stepwise route, in which the nitrogen-rich precursor is first associated with graphite before PMDA is introduced, produces materials with better-preserved diffraction features and more compact, layered morphologies than the corresponding one-pot route.

The microscopy results further show local perturbation of graphite stacking and the presence of nitrogen-rich material within graphite-containing particles, consistent with the close association of the two components. The combined evidence is therefore consistent with the reaction of preorganized melamine or melem in close spatial association with graphite and with at least partial formation of polyimide-linked material within the layered graphite-derived matrix. The data also indicate the presence of external growth and surface-associated domains, which is expected because excess melamine or melem remains outside the intercalated graphite regions before polycondensation.

## 4. Conclusions

This work demonstrates a solvent-free strategy for preparing graphite-based composites from melamine- and melem-derived precursors through mechanochemical preorganization followed by thermal transformation. The ball-milling conditions produced a graphite–melamine precursor capable of yielding few-layer graphene after melamine removal, confirming effective separation of the graphite layers. Intercalated melamine also retained the characteristic thermal reactivity of bulk melamine: annealing at 450 °C selectively arrested condensation at the melem stage, yielding a graphite–melem composite.

Melamine- and melem-derived polyimide reference systems were then optimized in the absence of graphite. PI(2:3)MA and PI(1:3)ME showed the clearest combination of imide formation and structural order and were selected for composite preparation. When these reactions were transferred to graphite-based precursors, the characteristic polyimide-linked phases remained detectable. The stepwise route generally produced better-preserved diffraction features and more compact, layered morphologies than the one-pot route, particularly for the melem-derived system.

FTIR confirmed retention of the characteristic imide chemistry in the graphite composites, while HRTEM and STEM–EDX revealed local perturbation of graphite stacking and the coexistence of graphite-rich and nitrogen-rich domains within the same particles. These results support close association and partial formation of polyimide-linked material within the layered graphite-derived matrix, together with external and surface-associated domains, although a regularly alternating interlayer architecture was not resolved. Overall, precursor intercalation, retained chemical reactivity, and stepwise preorganization provide a practical basis for constructing graphite-based composites with nitrogen-rich polyimide-linked materials.

## Data Availability

The data presented in this study are available on reasonable request from the corresponding author.

## Supplementary Materials

The following supporting information can be downloaded at: https://www.mdpi.com/article/10.3390/nano16161028/s1, Figure S1: Melamine-assisted exfoliation of graphite. (a) SEM of the dialyzed product, showing thin, wrinkled few-layer graphene. (b) TEM of electron-transparent flakes with folded edges from which the layer number is estimated; Figure S2: FTIR spectra of the MA-based series: PMDA, MA, PI(1:1)MA, PI(2:3)MA, PI(2:3)MA/G X, PI(1:1)MA/G X, PI(4:3)MA and PI(1:2)MA. Spectra are shown in transmittance and interpreted qualitatively (band positions and the presence/absence of characteristic bands); Figure S3: FTIR spectra of the ME-based series: ME, PI(1:1)ME, PI(2:1)ME, PI(1:2)ME, PI(1:3)ME, PI(1:4)ME and PI(1:3)ME/G X. Spectra are shown in transmittance and interpreted qualitatively; Figure S4: TEM images of the PI(1:3)ME, showing a hexagonal, plate-like morphology with well-defined edges. No lattice fringes were resolved, consistent with the low contrast and beam sensitivity of the organic framework; Figure S5: EDX elemental maps (O, green; C, red; N, blue) and the corresponding HAADF image of an individual PI(1:3)ME particle, showing a uniform distribution of carbon, nitrogen and oxygen. Scale bars: 200 nm; Figure S6: SAED patterns (top) and HRTEM lattice-fringe images (bottom) of the PI(2:3)MA/G X (left) and PI(1:3)ME/G X (right) composites. The SAED patterns show discrete diffraction spots from the crystalline graphitic phase; in the HRTEM images the graphite lattice fringes are locally less continuous near some edges; Figure S7: HRTEM image and corresponding FFT of the PI(2:3)MA/G X composite. The measured lattice spacings d = 0.35 ± 0.01 nm and d = 0.20 ± 0.01 nm are assignable to the graphite (002) interlayer and in-plane spacings, respectively; the ~0.35 nm value is also consistent with interlayer stacking of the polyimide-linked material and cannot be unambiguously distinguished from graphite; Figure S8: STEM–EDX elemental maps (HAADF; C, red; N, blue; O, green) of a graphite-rich region of the PI(2:3)MA/G X composite. The region is dominated by carbon with only a weak, scattered nitrogen signal, and the EDX spectrum is dominated by the carbon peak (the Cu signals arise from the support grid). Scale bars: 2 µm; Figure S9: STEM–EDX of a COF-containing region of the PI(1:3)ME/G X composite: HAADF image and C (red), N (blue) and O (green) maps (top), with point spectra acquired at two locations (bottom). Carbon and nitrogen are co-located within the particle, and the C:N ratio differs between the two locations. Scale bars: 100 nm; Figure S10: Domain-resolved STEM–EDX analysis of the PI(2:3)MA/G X composite. Top: elemental maps. Bottom: higher- and lower-magnification selected-area spectra with the corresponding quantification, showing that the C:N ratio differs significantly between different domains.

## Abbreviations

The following abbreviations are used in this manuscript:

G	Graphite
MA	Melamine
ME	Melem
PMDA	Pyromellitic Dianhydride
COF	Covalent Organic Framework
PI-COF	Polyimide Covalent Organic Framework
G+MA	Graphite–Melamine Precursor (obtained by ball milling)
G–ME	Graphite–Melem Composite (obtained via thermal transformation of G+MA)
G+ME	Graphite–Melem Mixture (obtained by direct ball milling of graphite with ME)
X route	Stepwise Processing Route
T route	One-pot Processing Route
SEM	Scanning Electron Microscopy
PXRD	Powder X-ray Diffraction
FTIR	Fourier Transform Infrared Spectroscopy
XPS	X-ray Photoelectron Spectroscopy
TEM	Transmission Electron Microscopy
HRTEM	High-Resolution Transmission Electron Microscopy
SAED	Selected Area Electron Diffraction
STEM	Scanning Transmission Electron Microscopy
EDX	Energy-Dispersive X-ray Spectroscopy
