# Supplementary material for: Confined Chemical Transformation of Melamine in Graphite Interlayers Toward Graphite-Based Composites with Nitrogen-Rich Two-Dimensional Materials
Source: Nanomaterials (Basel). 2026 Aug 19;16(16):1028. doi: 10.3390/nano16161028 (PMC13515982; doi:10.3390/nano16161028)
Supplement: Supplementary file 1 [file nanomaterials-16-01028-s001.zip › nanomaterials-4330397-supplementary.pdf]

Supplementary Materials

Confined Chemical Transformation of Melamine in Graphite Interlayers toward Graphite-Based Composites with Nitrogen-Rich Two-Dimensional Materials

Wei Zhou †, Haseeb Ur Rehman †, Zeming Wang, Oleksandr Ivasenko*

State Key Laboratory of Bioinspired Interfacial Materials Science, Institute of Functional Nano & Soft Materials (FUNSOM), Soochow University, Suzhou 215123, China.

*Correspondence: ivasenko@suda.edu.cn

† These authors contributed equally to this work.

Contents:

S1. Melamine-assisted exfoliation of graphite

Figure S1. Melamine-assisted exfoliation of graphite. (a) SEM of the dialyzed product, showing thin, wrinkled few-layer graphene. (b) TEM of electron-transparent flakes with folded edges from which the layer number is estimated.

S2. FTIR Analysis

Figure S2. FTIR spectra of the MA-based series: PMDA, MA, PI(1:1)MA, PI(2:3)MA, PI(2:3)MA/G X, PI(1:1)MA/G X, PI(4:3)MA and PI(1:2)MA. Spectra are shown in transmittance and interpreted qualitatively (band positions and the presence/absence of characteristic bands).

Figure S3. FTIR spectra of the ME-based series: ME, PI(1:1)ME, PI(2:1)ME, PI(1:2)ME, PI(1:3)ME, PI(1:4)ME and PI(1:3)ME/G X. Spectra are shown in transmittance and interpreted qualitatively.

S3. TEM Analysis

Figure S4. TEM images of the PI(1:3)ME, showing a hexagonal, plate-like morphology with well-defined edges. No lattice fringes were resolved, consistent with the low contrast and beam sensitivity of the organic framework

Figure S5. EDX elemental maps (O, green; C, red; N, blue) and the corresponding HAADF image of an individual PI(1:3)ME particle, showing a uniform distribution of carbon, nitrogen and oxygen. Scale bars: 200 nm

Figure S6. SAED patterns (top) and HRTEM lattice-fringe images (bottom) of the PI(2:3)MA/G X (left) and PI(1:3)ME/G X (right) composite. The SAED patterns show discrete diffraction spots from the crystalline graphitic phase; in the HRTEM images the graphite lattice fringes are locally less continuous near some edges

Figure S7. HRTEM image and corresponding FFT of the PI(2:3)MA/G X composite. The measured lattice spacings d = 0.35 ± 0.01 nm and d = 0.20 ± 0.01 nm are assignable to the graphite (002) interlayer and in-plane spacings, respectively; the ~0.35 nm value is also consistent with interlayer stacking of the polyimide-linked material and cannot be unambiguously distinguished from graphite.

Figure S8. STEM–EDX elemental maps (HAADF; C, red; N, blue; O, green) of a graphite-rich region of the PI(2:3)MA/G X composite. The region is dominated by carbon with only a weak, scattered nitrogen signal, and the EDX spectrum is dominated by the carbon peak (the Cu signals arise from the support grid). Scale bars: 2 μm.

Figure S9. STEM–EDX of a COF-containing region of the PI(1:3)ME/G X composite: HAADF image and C (red), N (blue) and O (green) maps (top), with point spectra acquired at two locations (bottom). Carbon and nitrogen are co-located within the particle, and the C:N ratio differs between the two locations. Scale bars: 100 nm.

Figure S10. Domain-resolved STEM–EDX analysis of the PI(2:3)MA/G X composite. Top: elemental maps. Bottom: higher- and lower-magnification selected-area spectra with the corresponding quantification, showing that the C:N ratio differs significantly between different domains.

S1. Melamine-assisted exfoliation of graphite

To verify that the ball-milling conditions used in this study promote melamine-assisted separation and exfoliation of graphite, few-layer graphene was prepared following a reported melamine-assisted exfoliation protocol [1]. Graphite and melamine were ball-milled at 30 Hz for 4 h. For the subsequent removal of residual melamine (MA), the composite powder was dispersed in deionized water under sonication, followed by dialysis under continuous stirring at 70 °C. Detailed experimental parameters, including the dialysis procedure, followed the previously reported protocol [1]. The exfoliated flakes were drop-cast onto a Si substrate for SEM imaging and a holey carbon-coated Cu grid for TEM imaging. SEM shows large, wrinkled, partially folded sheets with markedly reduced thickness relative to pristine graphite (Figure S1a), and TEM confirms electron-transparent flakes only a few layers thick, with folded edges giving a layer count of ~2–3 (Figure S1b). These results demonstrate effective melamine-assisted separation and exfoliation of the graphite layers, supporting the intercalation premise underlying the confined-transformation strategy.

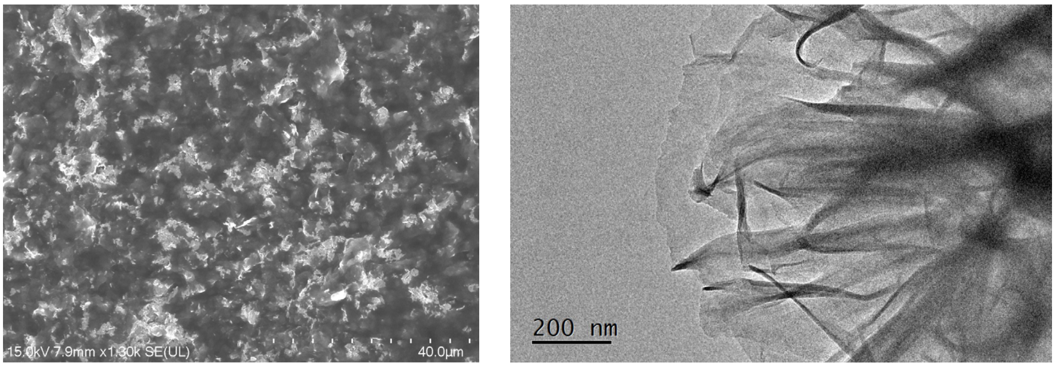

Figure S1. Melamine-assisted exfoliation of graphite. (a) SEM of the dialyzed product, showing thin, wrinkled few-layer graphene. (b) TEM of electron-transparent flakes with folded edges from which the layer number is estimated.

S2. FTIR Analysis

FTIR spectra were used to follow the formation of the polyimide-linked materials and their composites. All spectra were recorded in transmittance and are interpreted qualitatively; the assignments below are based on the positions and the appearance/disappearance of characteristic bands rather than on a quantitative comparison of band intensities.

For both the MA-based series (Figure S2) and the ME-based series (Figure S3), formation of the polyimide-linked products is indicated by the imide-related bands at ~1772, 1725, 1376 and 725 cm⁻¹, together with consumption of the N–H stretching bands (3100–3500 cm⁻¹) and loss of the anhydride band (~1850 cm⁻¹) of PMDA. The same diagnostic bands are retained in the corresponding graphite composites [PI(2:3)MA/G X, PI(1:1)MA/G X and PI(1:3)ME/G X], and no new bands appear upon compositing, indicating that graphite interacts predominantly through noncovalent association rather than through formation of new covalent bonds.

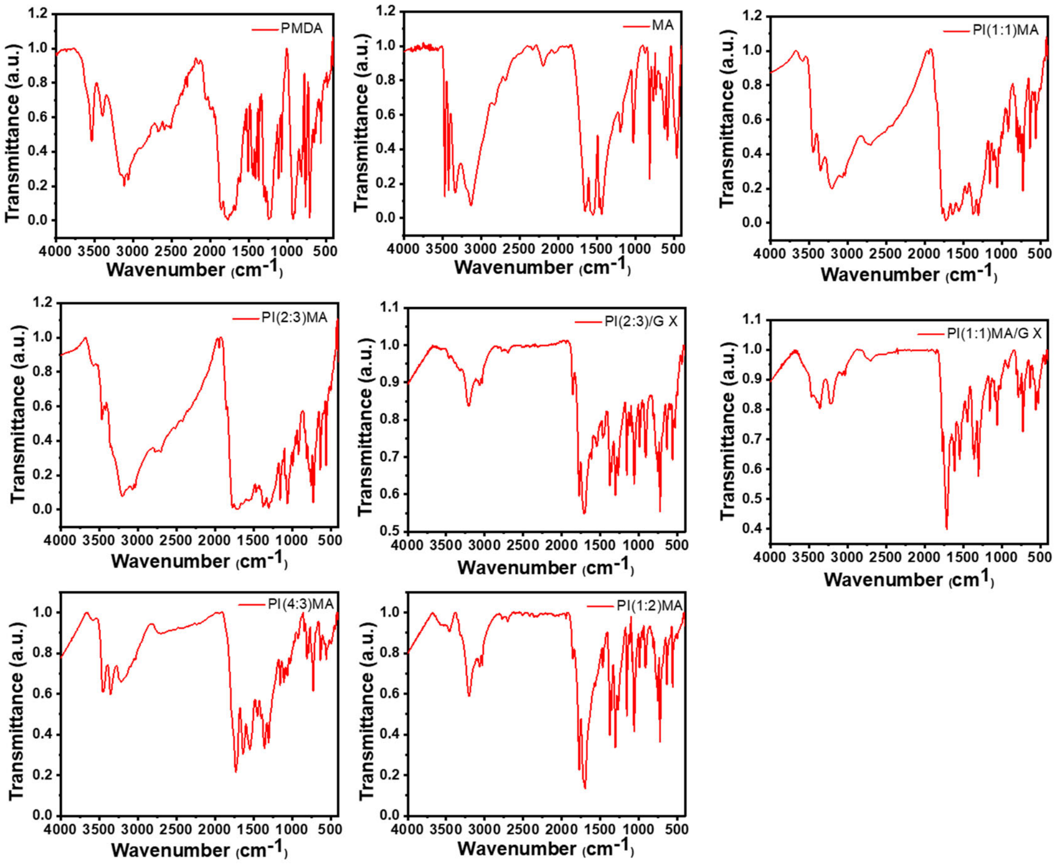

Figure S2. FTIR spectra of the MA-based series: PMDA, MA, PI(1:1)MA, PI(2:3)MA, PI(2:3)MA/G X, PI(1:1)MA/G X, PI(4:3)MA and PI(1:2)MA. Spectra are shown in transmittance and interpreted qualitatively (band positions and the presence/absence of characteristic bands).

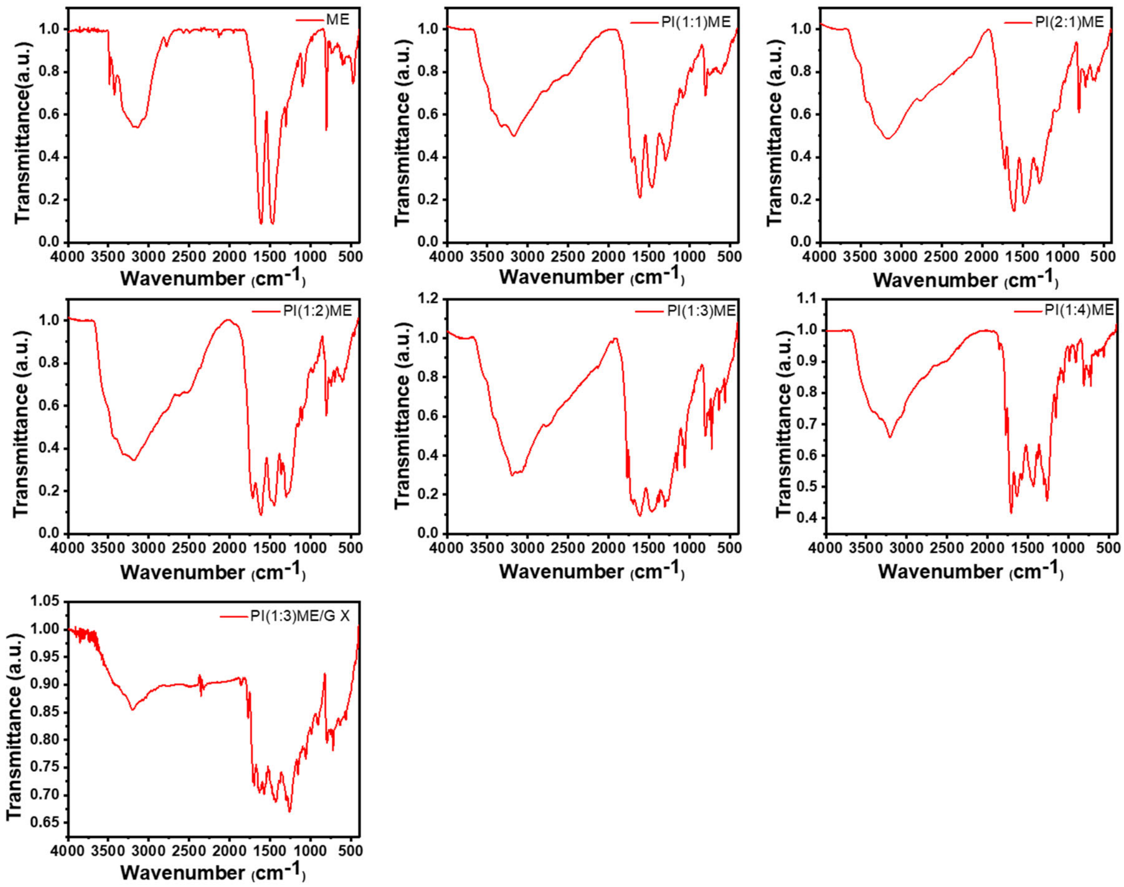

Figure S3. FTIR spectra of the ME-based series: ME, PI(1:1)ME, PI(2:1)ME, PI(1:2)ME, PI(1:3)ME, PI(1:4)ME and PI(1:3)ME/G X. Spectra are shown in transmittance and interpreted qualitatively.

S3. TEM Analysis

Transmission electron microscopy (TEM), high-resolution TEM (HRTEM), selected-area electron diffraction (SAED) and energy-dispersive X-ray spectroscopy (EDX) elemental mapping were performed on a Talos 200X microscope operated at 200 kV. Samples were gently dispersed in ethanol, briefly sonicated and drop-cast onto holey carbon-coated copper grids, then dried before imaging. Interlayer spacings were quantified from HRTEM lattice-fringe images using ImageJ.

The graphite-free PI(1:3)ME reference material was first examined (Figures S4 and S5). The PI(1:3)ME particles exhibit a hexagonal, plate-like morphology with well-defined edges (Figure S4); however, no lattice fringes could be resolved, consistent with the low electron-density contrast and beam sensitivity of the organic framework. EDX elemental mapping of an individual COF particle (Figure S5) shows that carbon, nitrogen and

oxygen are distributed uniformly across the particle, as expected for the graphite-free polyimide-linked material.

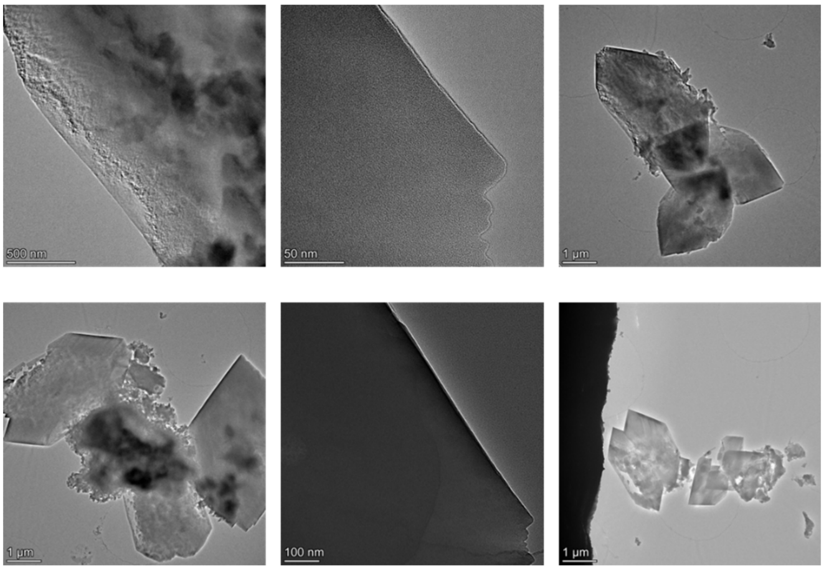

Figure S4. TEM images of the PI(1:3)ME, showing a hexagonal, plate-like morphology with well-defined edges. No lattice fringes were resolved, consistent with the low contrast and beam sensitivity of the organic framework.

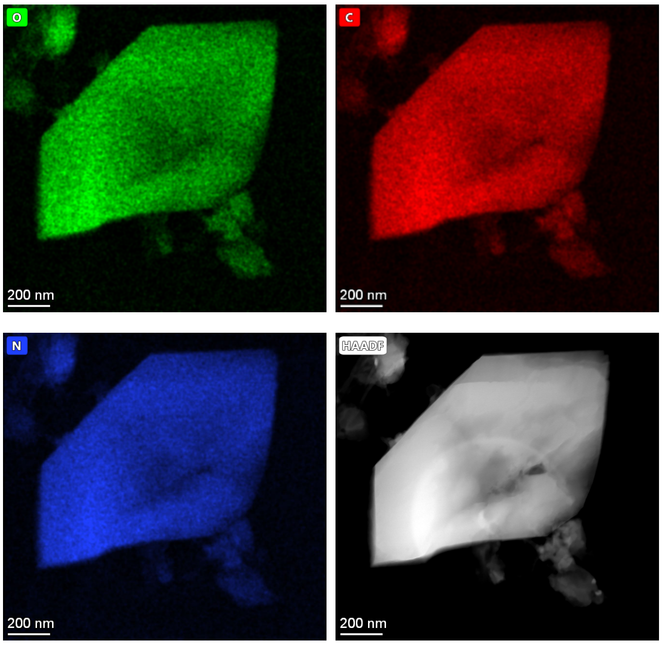

Figure S5. EDX elemental maps (O, green; C, red; N, blue) and the corresponding HAADF image of an individual PI(1:3)ME particle, showing a uniform distribution of carbon, nitrogen and oxygen. Scale bars: 200 nm.

HRTEM of the PI(2:3)MA/G X and PI(1:3)ME/G X composites reveals the graphite lattice fringes; in some edge regions these fringes become less continuous and more dispersed than in pristine graphite, indicating a local perturbation of the layer stacking (Figure S6). Quantitative analysis of the lattice fringes gives an average spacing of ~0.35 nm, slightly larger than the ~0.335 nm spacing of pristine graphite and consistent with modest local expansion of the layered structure. Lattice-resolved imaging and the corresponding fast Fourier transform (FFT) reveal periodic features with spacings of ~0.35 nm and ~0.20 nm (Figure S7), assignable to the graphite (002) interlayer and in-plane spacings, respectively. We note that the ~0.35 nm spacing is close to the expected interlayer stacking of the polyimide-linked material, so the two cannot be unambiguously distinguished and no separate COF reflection was resolved. SAED (Figure S6) shows discrete diffraction spots confirming the crystallinity of the graphitic phase.

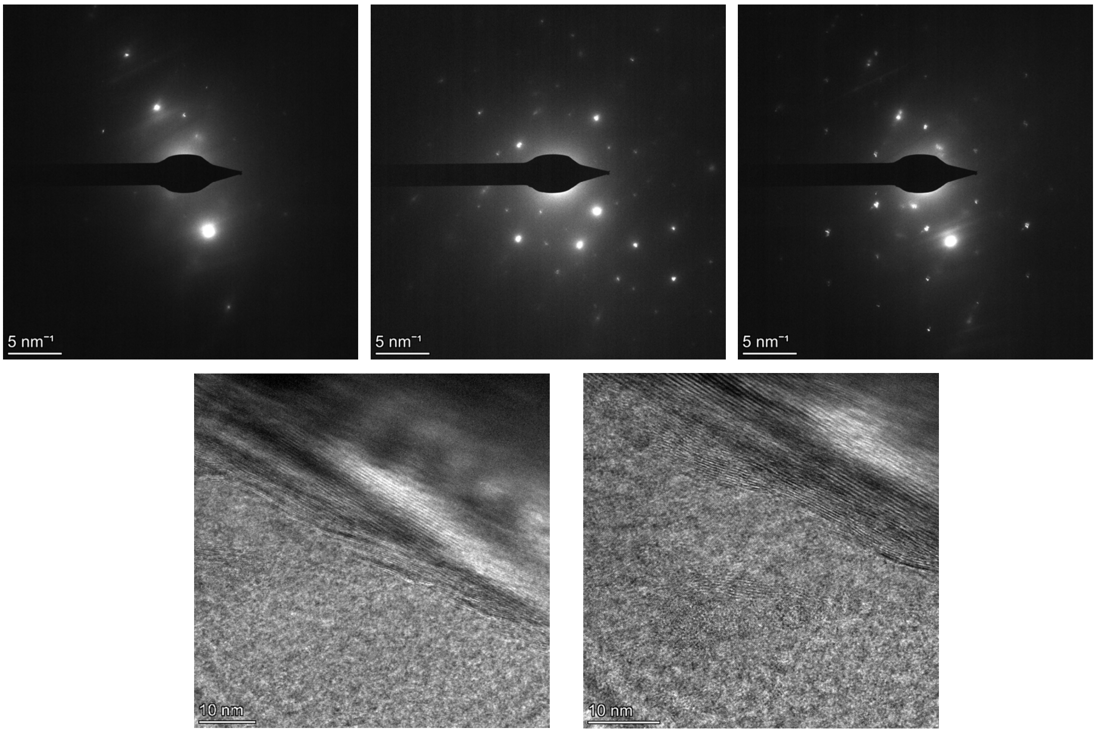

Figure S6. SAED patterns (top) and HRTEM lattice-fringe images (bottom) of the PI(2:3)MA/G X (left) and PI(1:3)ME/G X (right) composites. The SAED patterns show discrete diffraction spots from the crystalline graphitic phase; in the HRTEM images the graphite lattice fringes are locally less continuous near some edges.

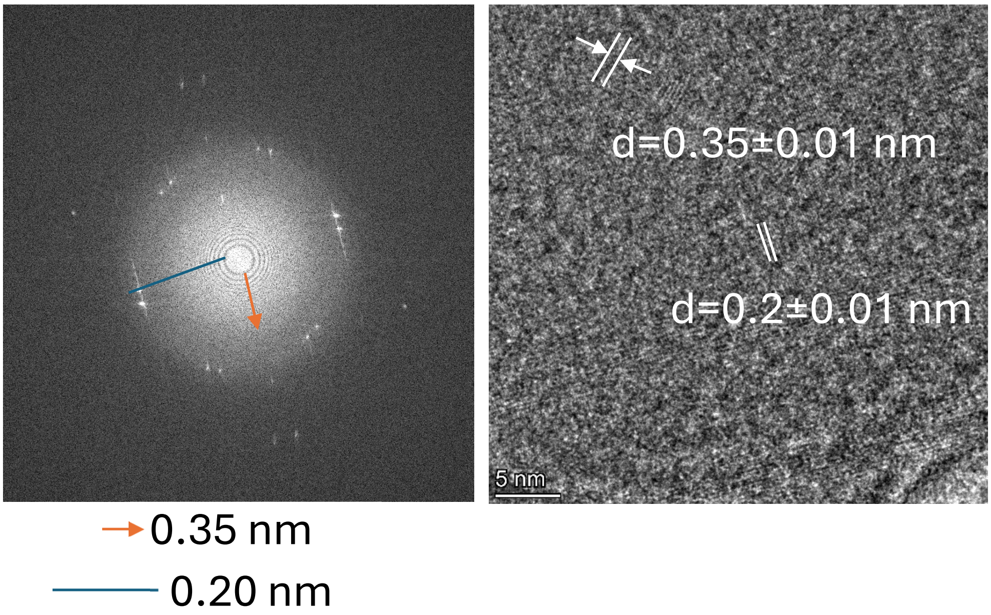

Figure S7. HRTEM image and corresponding FFT of the PI(2:3)MA/G X composite. The measured lattice spacings d = 0.35 ± 0.01 nm and d = 0.20 ± 0.01 nm are assignable to the graphite (002) interlayer and in-plane spacings, respectively; the ~0.35 nm value is also consistent with interlayer stacking of the polyimide-linked material and cannot be unambiguously distinguished from graphite.

EDX elemental mapping was used to probe the spatial distribution of carbon and nitrogen in the composites (Figures S8–S10). The composite is compositionally heterogeneous: some regions are graphite-rich, showing a strong carbon signal with only weak nitrogen (Figure S8), whereas other regions contain substantial nitrogen together with carbon within the same particles (Figure S9). Point spectra acquired at different locations (Figure S9) and layer/domain-resolved STEM–EDX analysis (Figure S10) confirm that the C:N ratio varies markedly between different domains of the composite. Where nitrogen is present, it is distributed throughout the projected particle area rather than confined to the outer surfaces (Figure S9), consistent with incorporation of the nitrogen-rich (COF-derived) component within the composite rather than as a purely external coating. We note that, at the present spatial resolution, the carbon and nitrogen signals are largely co-located within the same domains; the EDX data therefore evidence intimate mixing and domain-level compositional variation between graphite-rich and COF-rich regions, rather than cleanly resolved, alternating carbon and nitrogen layers.

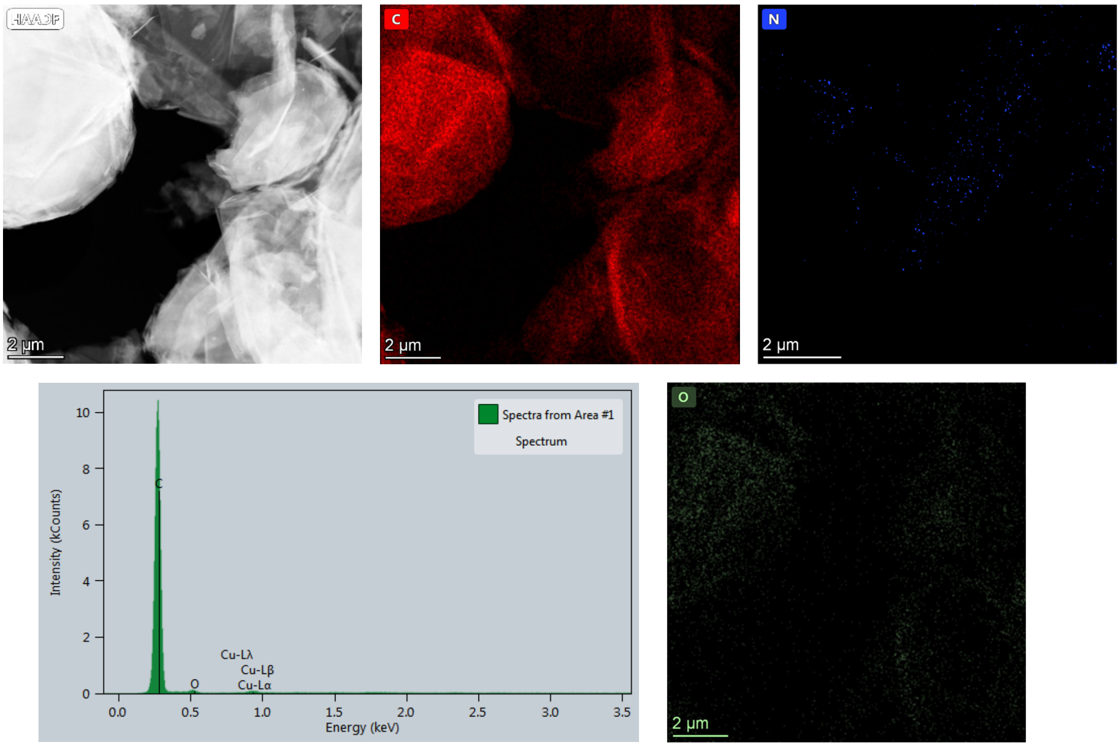

Figure S8. STEM–EDX elemental maps (HAADF; C, red; N, blue; O, green) of a graphite-rich region of the PI(2:3)MA/G X composite. The region is dominated by carbon with only a weak, scattered nitrogen signal, and the EDX spectrum is dominated by the carbon peak (the Cu signals arise from the support grid). Scale bars: 2 μm.

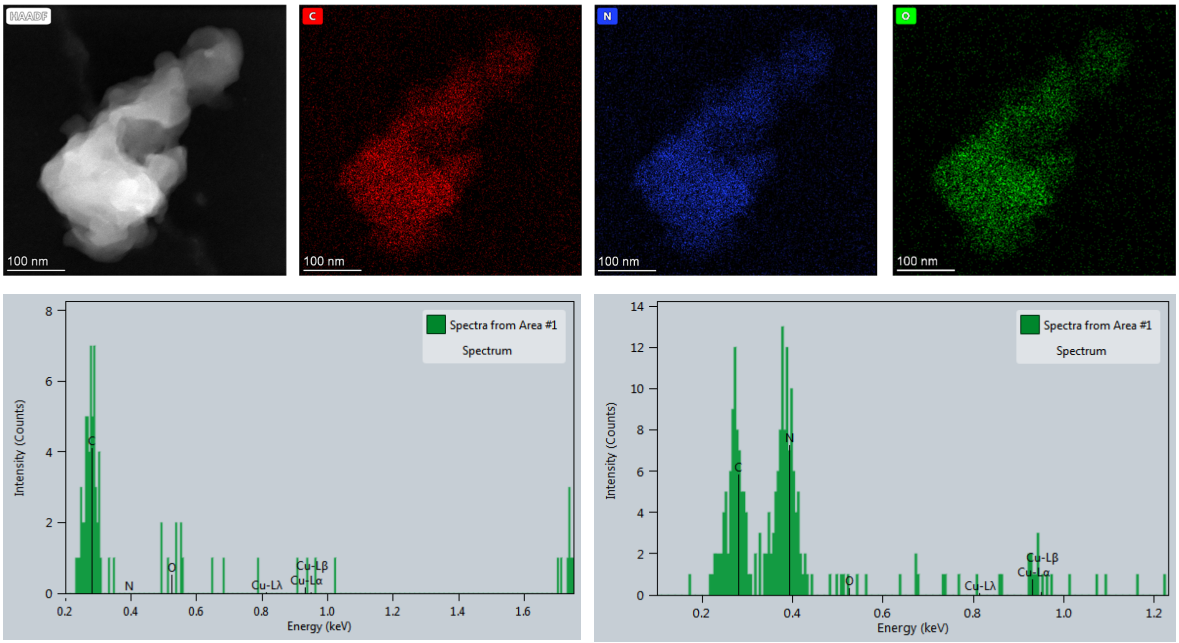

Figure S9. STEM–EDX of a COF-containing region of the PI(1:3)ME/G X composite: HAADF image and C (red), N (blue) and O (green) maps (top), with point spectra acquired at two locations (bottom). Carbon and nitrogen are co-located within the particle, and the C:N ratio differs between the two locations. Scale bars: 100 nm.

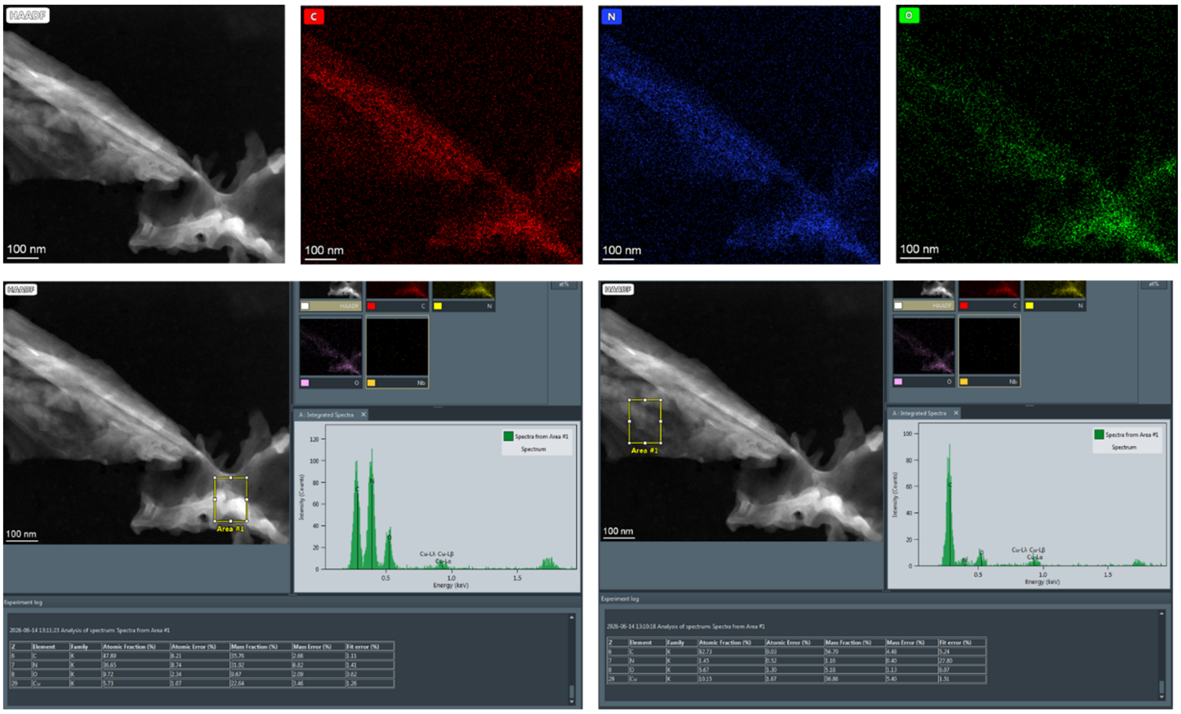

Figure S10. Domain-resolved STEM–EDX analysis of the PI(2:3)MA/G X composite. Top: elemental maps. Bottom: higher- and lower-magnification selected-area spectra with the corresponding quantification, showing that the C:N ratio differs significantly between different domains.

References

1. González-Domínguez, J.M.; León, V.; Lucío, M.I.; Prato, M.; Vázquez, E. Production of Ready-to-Use Few-Layer Graphene in Aqueous Suspensions. *Nat Protoc* **2018**, *13*, 495–506, doi:10.1038/nprot.2017.142.
